# Childbirth Acquired Perineal Trauma study (CHAPTER): a UK prospective cohort study protocol

**DOI:** 10.1136/bmjopen-2024-086724

**Published:** 2024-05-24

**Authors:** Victoria Hodgetts Morton, Rebecca Man, Rita Perry, Terry Hughes, Susan Tohill, Christine MacArthur, Laura Magill, R Katie Morris

**Affiliations:** 1 Birmingham Women's Hospital, Birmingham, UK; 2 Institute of Applied Health Research, University of Birmingham, Birmingham, UK

**Keywords:** Postpartum Period, Natural Childbirth, Postpartum Women, OBSTETRICS, Pregnancy, WOUND MANAGEMENT

## Abstract

**Introduction:**

Childbirth-related perineal trauma (CRPT) is the most common complication of childbirth affecting 80% of women overall after vaginal birth. There remains a lack of comprehensive evidence relating to the prevalence of subsequent health problems. Current evidence is related to short-term outcomes, for example, pain, but there is less known about longer-term outcomes such as infection, wound dehiscence, pelvic floor function and psychological outcomes. This is a protocol for a cohort study assessing outcomes of women after CRPT.

**Methods and analysis:**

A multicentre, prospective UK cohort study aiming to include 1000 women. All women who have sustained CRPT will be eligible for inclusion and will be followed-up for 12 months after childbirth. The primary outcome will be perineal infection at 6 weeks post-birth. Secondary outcomes will include antibiotic use for perineal infection, wound breakdown, use of analgesia, the requirement for admission or surgical intervention, urinary and faecal incontinence, anxiety and depressive symptoms, sexual function and impact on daily activities. Outcomes will be measured at 6 weeks, 6 months and 12 months post partum, with some outcomes being measured at all time points and others at selected most appropriate time points only. Outcome data will be obtained from a review of clinical notes and from patient questionnaires. Simple descriptive statistics will be used to summarise characteristics and outcomes, with categorical variables expressed as percentages and continuous variables as mean averages, alongside the corresponding standard deviatons.

**Ethics and dissemination:**

Ethical approval has been granted by the Research Ethics Council with reference 23/WA/0169. Data collected from the Childbirth Acquired Perineal Trauma (CHAPTER) cohort study will highlight the prevalence and type of complications after CRPT and which women are more at risk. After the conclusion of this study, findings will be used to work with governmental organisations and Royal Colleges to target resources and ultimately improve care.

**Strengths and limitations of this study**

- This study aims to incorporate a diverse range of women with childbirth-related perineal trauma (CRPT) from across the UK, with the goal of producing representative and up-to-date information on complications after CRPT.
- Follow-up questionnaires are comprehensive and holistic with a range of potential physical and psychological complications after CRPT being assessed.
- Follow-up for the year after childbirth is integral to this study and we will need to consider the potential for non-response to the questionnaires, particularly at the later data collection points.
- Questionnaires will need to be completed and returned within the specified time frame in relation to the follow-up points. Where responses occur outside these time windows we will need to consider the impact of this on response.

## Introduction

In the UK every year, 80% of women who have a vaginal birth (approximately 450 000 women), experience childbirth-related perineal trauma (CRPT).^1 2^ CRPT affects 91% of women in a first birth and 70% in successive births and is therefore the most common complication after childbirth.^2^ Despite CRPT affecting the vast majority of women who give birth, the prevalence and severity of resulting health complications remain unclear. There is some evidence relating to initial outcomes, for example, pain, but less is known about longer-term complications, including; wound dehiscence, infection, psychological or social issues, sexual dysfunction and incontinence.^3–6^

Complications from CRPT can be multifaceted and long-lasting, with some women suffering wound-related complications for many years and ultimately requiring complex reconstructive surgery, or developing long-term psychological problems.^7 8^ There is currently very little research exploring the prevalence of severe CRPT, its risk factors, management or its short or long-term complications.^9^ Information to tell us how serious these complications are or how often they occur, is not routinely collected. There is very little research to indicate which women might be more likely to suffer complications from CRPT or experience longer-term health problems.^3–6^ The Childbirth Acquired Perineal Trauma (CHAPTER) cohort study is designed to address this substantial evidence gap.

This multicentre, prospective, UK-based cohort study will follow a diverse group of UK women who have sustained CRPT. We will determine the current assessment and management of CRPT immediately after it is sustained, in addition to the prevalence and type of short-term and long-term complications in women who have CRPT. Risk factors for developing CRPT complications and current practice of assessment and management of CRPT complications will be ascertained.

## Methods and analysis

### Objectives

- To describe the current assessment and management of CRPT at the time it is sustained.
- To determine the prevalence of short-term and long-term complications or health problems in women who have CRPT.
- To describe the management of subsequent complications in women with CRPT.
- To describe the risk factors associated with the development of CRPT-associated complications or longer-term sequelae.

### Study design

A UK multicentre prospective cohort study.

### Study setting

CHAPTER is open to any type of unit in the UK which offers maternity care. This includes obstetrical-led, alongside midwifery units and freestanding midwifery units.

### Sample size calculations

We will aim to recruit a minimum of 1000 women. 300 participants will allow us to determine a CRPT complication prevalence rate of 10% with a 95% CI of 6.8% to 14.0%. A sample size of 900 will allow rarer outcomes to be estimated with precision, for example, a 95% CI for an estimate of 5% would range from 3.7% to 6.6%. We anticipate the sample will include approximately 450 women in the minor trauma group and 450 women in the second, third and fourth degree tear group. We have in excess of 99% power to detect the difference in pain relief previously seen and with the anticipated sample we would be able to detect a difference of less than 10% points between groups (25% vs 35%).

Approximately 70% of women have a vaginal delivery (55–60% unassisted; 10–15% assisted) and there are 73 889 live births per 6-week period, thus 51 722 with a vaginal delivery of which 39 723 will sustain non-obstetric anal sphincter injury (non-OASI) CPRT and 2068 OASI CRPT.^1 10^ Based on 20 maternity sites participating (n=9930) and 80% of those being suitable to be approached (eligible, baby or mother not too unwell to be approached—7944), if 50% of those eligible are successfully approached with 50% consenting (1986) and 50% completing all follow-up gives a potential sample of n=993 for all outcomes. For those outcomes that can be determined by routine data collection, this gives a potential sample of n=1986.^11 12^

### Inclusion criteria

To include women who fit any of the following criteria:

- Women (≥16 years) who have given birth either spontaneously or operatively and sustained CRPT (including OASI) who are able and willing to give informed consent.
  - To include women who have failed instrumental delivery who ultimately give birth via caesarean section provided they have sustained CRPT.
  - To include women with multiple births who give birth vaginally and via caesarean section provided they have sustained CRPT.

### Exclusion criteria

None.

### Outcomes to be measured

Primary outcome: perineal infection within 6 weeks post-birth.

Secondary outcomes:

#### Clinical outcomes from medical records check

- Antibiotic use for perineal infection.
- Wound breakdown.
- Urinary and faecal incontinence.
- Readmission or triage visit for CRPT-related complications.
- Requiring referral or review within a specialist perineal clinic.
- Requiring minor or major corrective perineal surgery.

#### Patient-reported outcomes as obtained from questionnaires

At 6 weeks, 6 and 12 months post partum:

- Urinary incontinence via the Revised Urinary Incontinence Scale.^13^
- Faecal incontinence via the Revised Faecal Incontinence Scale.^14^
- Anxiety and depression via the Edinburgh Postnatal Depression Scale.^15^
- General health-related quality of life measured using the EQ-5D-5L tool (EuroQol 5 dimension, 5 level).^16^
- Post-traumatic stress disorder (PTSD) via selected domains from the City Birth Trauma Scale^17^
- Measures of pain.
- Readmissions.

At 6 and 12 months post partum only:

- Sexual function via the Bespoke CHAPTER Questionnaire.
- Physical activity via the Pelvic Floor Impact Questionnaire.^18^
- Pelvic organ prolapse symptoms via selected domains from the Revised Postpartum Pelvic Floor and Birth Questionnaire.^19^
- Maternal satisfaction.
- Ability to feed baby according to mother’s preference.
- Ability to care for baby.
- Social isolation
- Ability to care for older children.
- Impact on seeking healthcare.
- Future pregnancy and subsequent mode of birth preference.

### Identification of participants and recruitment

Potentially eligible participants will be identified by members of the clinical care or research team at the relevant site. This will include midwives and obstetricians (consultants and trainees), research midwives and nurses in maternity units in secondary and tertiary care NHS Trusts. All participants screened for inclusion in the CHAPTER study will be recorded on the online CHAPTER screening log. No identifiable information will be included on the screening log. This log will be prospectively maintained at each site using either the hospital birth register or the postnatal ward-specific list of births. The CHAPTER screening log will be held on the Research Electronic Data Capture (REDCap) system.

There will be a maximum of three defined 14-day data collection periods. We shall review demographics and type of CRPT after each 14-day data collection period to ensure that we are achieving representation related to non-OASI and our aim for inclusivity. This will allow us to purposively recruit in subsequent rounds if required.

### Consent process

Within the CHAPTER cohort study, two different methods can be used to obtain informed consent for the study:

- In-person written informed consent.
- Remote consent.

Irrespective of the type of consent to be used, the first approach to potential participants for possible inclusion in the study must be made by a member of the clinical care team at the site (eg, midwife or obstetrician).

The initial approach to potential participants will, in most cases, take place while women are still an inpatient and can be in one of two settings:

- On the labour ward/birthing unit for those women anticipating an early discharge but at least 6 hours after birth.
- On the postnatal ward.
- For those women who have a home birth or who are discharged before they have been approached for inclusion, they will be asked to participate when they are seen by a community midwife usually on day 1 post partum.

Women will be provided with background information about the study, including the premise and what their participation would involve. The women will have an opportunity to ask questions and a written CHAPTER study patient information sheet (PIS) will be provided as an adjunct to this conversation.

Those for whom English is not their first spoken language will also be supported by standard Trust interpretation services, for example, Trust interpreters or LanguageLine (a UK language translation service agency that provides interpreting and translation services in over 200 languages, including British Sign Language). Women who understand spoken English will be able to participate in the study. They will be contacted via telephone and asked to complete the follow-up questionnaires over the telephone with a translator should they choose to. A record of the discussion with the women will be kept in their medical notes.

#### Remote consent process

Remote documented consent is an option within the CHAPTER study. Remote consent may be undertaken if in-person consent is not feasible, for example, in areas where participants are likely to have a home birth due to the geographical area. In these instances, the woman will be provided with the PIS and consent form by the community midwife or by post. The informed consent discussions will proceed as detailed, but by telephone or video-conference and the details will be recorded in the patient’s medical notes.

There are then two options that may be used to document informed consent. The choice of which method is the decision of the research team at the site:

- Scanned/photograph of consent form:

The patient will be asked to initial the boxes on the consent form, and sign and date the form while on the call. If the consent form was sent to the patient by post the patient will be asked to take a photo or scan of the signed form, and email it to the site staff, who will print it off and counter-sign. If the consent form was sent to the patient electronically, and they have access to a printer, they can print a hard copy, take a photo or scan of the signed form and email it to the site staff, who will print it off and counter-sign. The original signed and counter-signed consent form will be placed in the Investigator Site File (ISF), a copy will be sent to the participant (electronically or by post) and a copy will be filed in the patient’s medical notes.

- Postal consent:

The patient will be asked to initial the boxes on the consent form, and sign and date the form while on the call. The patient should then post the signed consent form to the site staff, who will counter-sign it on receipt. The original-signed and counter-signed consent form will be placed in the ISF, a copy will be posted to the participant and a copy will be filed in the patient’s medical notes.

### Withdrawal of consent

Participants will be made aware from the beginning of their involvement in the study that they can freely withdraw at any time. Continued consent will be assumed by the completion and return of the questionnaires at each time point. Participants will be given details on how to withdraw from the study at any point in the PIS. Reasons for withdrawal must be recorded on the CHAPTER REDCap database.

If a participant decides that they do not wish to continue their participation in the CHAPTER study, they may withdraw in one of two ways:

1. No study-related follow-up: The participant does not wish to complete patient-reported study-specific follow-up (ie, patient-reported outcomes and quality of life measures at 6 weeks, 6 and 12 months post partum) but is willing for routinely collected data to be used for inclusion in the study.
2. No further data collection: The participant is not willing to be followed-up in any way for the purposes of the study AND does not wish for any further data to be collected (ie, only data collected prior to withdrawal can be used in the study analysis).

If a participant withdraws from the CHAPTER study, their study record on the CHAPTER REDCap database will be closed and no further data will be collected, as per the participant’s request. All data collected until that time point, will be retained and included in the analysis.

### Participant registration

Registration will be via a secure online system hosted by the University of Birmingham Centre for Prospective and Observational Studies (BiCOPS); www.CHAPTER-bicops.bham.ac.uk. Unique log-in usernames will be provided to those who wish to use the online system and who have been delegated the role of registering participants in the study.

After eligibility for registration has been confirmed and informed consent has been taken and documented, the participant can be registered to the study using the CHAPTER online system. During the online registration process, eligibility must be confirmed and participant contact details for follow-up must be entered. A registration number will be given once all questions have been answered. Following registration, a confirmatory email will be sent to the responsible clinician, midwife and local principle investigator (PI). The local research team should add the participant to the CHAPTER participant recruitment and identification log which links participants with their CHAPTER study number. PIs must maintain this document securely and it must not be submitted to the study office. The CHAPTER participant recruitment and identification log should be held in strict confidence.

Once the patient has consented to entry into CHAPTER, their General Practitioner should be notified that they are in the CHAPTER study, using the CHAPTER study GP letter. No other parties outside of the study team will be informed of the participant’s entry into the study.

### Baseline data collection

After the woman has consented and they are registered on to the CHAPTER study, baseline information will be collected (outline of information collected in table 1). Information will be collected from the maternity record related to demographics (eg, age, ethnicity), maternal characteristics (eg, body mass index, obstetrical history, medical conditions) and delivery characteristics (eg, mode of delivery, CRPT type and details of repair). There are no additional study-specific follow-up hospital visits once the participant is entered into the CHAPTER study.

**Table 1:** A summary of baseline study participant characteristics

Category	Baseline data item
Demographic information	Age
	Ethnicity
	Geographical location
Maternal characteristics	Booking weight
	Body mass index
	Comprehensive obstetrical history including previous tear details
	Medical conditions pre-existing or during pregnancy
	Smoking status
	Female genital mutilation status, type and deinfibulation
Labour and birth details	Onset of labour: spontaneous or induced
	Length of second stage
	Singleton/multiples birthed
	Mode and setting of delivery
	Presentation and position at birth
	Birth outcome: live birth/stillbirth
	Episiotomy
	Hands on/poised/off perineum at crowning
	Maternal position
	Shoulder dystocia and management if occurred
	Blood loss
	Analgesia used in labour
	Presence of chorioamnionitis
	Use of antibiotics in labour or immediately post partum and indication
	Baby details: gestation, sex, weight and measurements
Tear details, repair details	Grade and role of clinician who inspected perineum after birth
	Classification of tear: first-degree tear, second-degree tear, third-degree tear, fourth-degree tear, episiotomy, other lacerations
	Repair performed: method, suture material, by whom, in which setting
	Analgesia during and after repair
	Examinations performed before/after repair (vaginal examination/per rectal examination) and outcome
	Did baby stay with mother during repair?
	Was follow-up of tear arranged?

### Data collection time points

All follow-up will be conducted remotely. At the time of study entry, participants will be asked to specify their preferred method of contact. Participants will be contacted at the follow-up time points, using their preferred method of communication where possible to request completion of the CHAPTER study questionnaire (online supplemental appendix S1—6-week questionnaire). There are three follow-up time points at 6 weeks, 6 months and 12 months post partum.

10.1136/bmjopen-2024-086724.supp1 Supplementary data

At 6 weeks post partum: A clinical note review of electronic secondary care records will be undertaken. This combined with the questionnaire data at this time point, will be used to determine whether the primary outcome of perineal infection within 6 weeks post partum has been met.

At all time points, participants will be asked to complete questionnaire items relating to: Urinary incontinence, flatus incontinence, faecal incontinence, anxiety and depressive symptoms, general health-related quality of life, PTSD symptoms (please see outcomes section for specific questionnaires used).

At 6 and 12 months post partum only: participants will be asked questions relating to sexual function, physical activity, pelvic organ prolapse, maternal satisfaction, ability to feed the baby according to mother’s preference, ability to care for baby, social isolation, ability to care for older children (if relevant), impact on seeking healthcare, future pregnancy and mode of birth preference.

Where questionnaires indicate that a woman had to seek medical care related to her CRPT, further data will be collected by reviewing the secondary care medical records.

Independent data validators will assess a proportion of the data from included to assess generalisability, inclusivity and data accuracy via validation of the primary outcome and critical data items.

### Safety considerations and assessment of risk

CHAPTER is a cohort study, therefore any interventions that CHAPTER participants undergo will be as part of their routine clinical care. For this reason, there are no safety considerations relating to the study. We do not anticipate any risks to study participants. Enrolment in the study will not have any impact on patient care as participants will be receiving the same treatment that they would if they were not enrolled. There is a potential benefit for participants in that contact with a member of the research team may identify a woman experiencing concerning symptoms and/or signs of a physical or mental illness. Any women identified through questionnaires as being particularly distressed will be contacted and offered support by the research team with signposting to appropriate local services. Safeguarding elements related to significant concerns related to physical or mental health will involve contact with local services (primary and/or secondary care as appropriate).

Study participants may find the follow-up contacts for completion of the questionnaires an inconvenience given that the follow-up period continues up to 12 months post-study entry. We will mitigate any potential risk of attrition bias by ensuring that participants fully understand the time commitment at enrolment stage and that they know they may withdraw at any time. In accordance with the University of Birmingham’s standard operating procedures, this study has been risk assessed to clarify any risks relating uniquely to this study beyond that associated with usual care. A risk assessment has been conducted and concluded that this study presents no higher risk than the risk of standard medical care.

Women and clinicians may become distressed when completing the questionnaires. We have therefore developed care pathways within the study to manage this (online supplemental appendix S2).^20^ For participants who have completed the questionnaires electronically, we will ask them if they have found the questionnaires distressing and would like additional support from the CHAPTER team. Should participants indicate distress and would like a telephone call, they will be contacted within 7 days and a member of the team will direct them towards appropriate postnatal support services and their GP. If a woman experiences distress while completing the questionnaires with the clinician over the telephone she will be offered an opportunity to stop the questionnaire, or postpone to a later point and directed to support services as above. Should the clinicians conducting the telephone questionnaires, who are trained midwives/obstetricians experienced in the care of postnatal women with perineal trauma, become distressed by women’s experiences and/or symptoms, they will be offered support within the CHAPTER team through the chief investigator, in keeping with the normal clinical pathways for doctors and midwives working in the National Health Service.

10.1136/bmjopen-2024-086724.supp2 Supplementary data

### Data management

Data entry will be completed by the sites via a bespoke BiCOPS REDCap study database. The data capture system will conduct automatic range checks for specific data values to ensure high levels of data quality. Queries and requests for missing clinical data will be raised using data clarification forms via the study database, with the expectation that these queries will be completed by the site within 30 days of receipt.

Participant questionnaires will be completed online, by post or telephone according to participant preference. At the time of consent and entry into CHAPTER, what the study involves will be discussed with each woman. It will be explained that completion of quality-of-life questionnaires plus other patient-reported outcomes will be requested until 12 months post partum and the methods of completion of the questionnaires discussed. Participants who have not returned completed forms will be contacted up to three times after the due date to request completion of the questionnaires. After this time, unreturned forms will be regarded as missing and will not be requested again. At the 6-week time point, participant questionnaires can be completed at 6 weeks post partum, ±1 week. At the 6 and 12-month time points, participant questionnaires can be completed ±1 month.

The University of Birmingham has policies in place, which are designed to protect the security, accuracy, integrity and confidentiality of personal data. The study is registered with the Data Protection Officer at the University of Birmingham and will hold data in accordance with the Data Protection Act (2018 and subsequent amendments). The CHAPTER study office has arrangements in place for the secure storage and processing of the study data which comply with University of Birmingham policies.

It is the responsibility of the PI to ensure all essential study documentation and source documents (eg, signed consent forms, ISFs, participants’ hospital notes) at their site are securely retained for the contractual period. Archiving will be authorised by BiCOPS following the submission of the end-of-study report. No documents should be destroyed without prior approval from the BiCOPS Director. The electronic study master file will be stored at BiCOPS for at least 3 years after the end of the study. Long-term offsite data archiving facilities will be considered for storage after this time; data will be stored securely and confidentially for at least 25 years.

### End of study definition

The end of study will be when the last enrolled participant completes their 12-month questionnaires, all database queries have been resolved and the database has been locked and analyses completed. The CHAPTER study office will notify the Research Ethics Council (REC) and the sponsor within 90 days of the end of study. The CHAPTER study office will provide the REC and the sponsor with a summary report of the study.

### Data analyses and statistical considerations

Summary statistics: Simple descriptive statistics will be used to summarise characteristics and outcomes, with categorical variables expressed as percentages and continuous variables as mean averages alongside the corresponding standard deviation. To allow for hospital variation, differences in primary and secondary outcomes by participant characteristics will be assessed using multilevel logistic and linear regression models; unadjusted and adjusted models, using clinically plausible explanatory variables, will be reported. Further analysis will investigate potential subgroups, identified from our ongoing systematic reviews.

Explanatory variables/characteristics: We plan to explore the risk of our outcomes adjusted for variables deemed potentially significant. These will be identified in the following ways: other work packages within the programme grant related to primary care data and systematic reviews of existing literature, patient and public involvement and clinical co-applicants.

### Status and timeline of study

The CHAPTER cohort study started participant recruitment on 1 September 2023 and has undergone two rounds of recruitment to date (correct as of 19 March 2024). Questionnaires have followed at the initial 6-month time point and are currently being distributed at the 6-month time point. The 12-month questionnaires will follow accordingly from 1 September 2024 onwards.

### Data availability

Requests for data generated during this study will be considered by BiCOPS. Data will typically be available 6 months after the primary publication. Only scientifically sound proposals from appropriately qualified research groups will be considered for data sharing. The request will be reviewed by the BiCOPS Data Sharing Committee in discussion with the chief investigator and, where appropriate (or in the absence of the cheif investigator) any of the following: the study sponsor, the study management group, and the programme steering committee. A formal data sharing agreement (DSA) may be required between respective organisations once the release of the data is approved and before data can be released. Data will be fully de-identified (anonymised) unless the DSA covers the transfer of participant-identifiable information.

### Patient and public involvement statement

Patient and public involvement has been key to the CHAPTER project. We have a patient advisory group (PAG) who have been providing guidance on the development and initiation of this cohort study. The PAG have been instrumental in developing study materials, ethical considerations and in guiding decisions about rounds of recruitment needed. The PAG will continue to guide all aspects of the CHAPTER project development.

## Ethics and dissemination

Ethical approval has been granted by the REC with reference 23/WA/0169. It is anticipated that minor amendments may need to be submitted after the commencement of the study. These will be dealt with via submission to the ethics board and subsequently communicating this amendment to sites to ensure their consent is in place.

Results of the study will ultimately be submitted for publication in a peer-reviewed journal after study completion. Findings will also be submitted to national and international conferences as well as disseminated to the Department of Health and Royal Colleges. Appropriate resources for patients will be developed and any relevant findings are expected to be rapidly incorporated into guidelines by the Royal College of Obstetricians and Gynaecologists, the Royal College of Midwives and the National Institute for Health and Care Excellence.

## Supplementary Material

Reviewer comments

Author's manuscript
