# Supplementary material for: Childbirth Acquired Perineal Trauma study (CHAPTER): a UK prospective cohort study protocol
Source: BMJ Open. 2024 May 24;14(5):e086724. doi: 10.1136/bmjopen-2024-086724 (PMC11129024; doi:10.1136/bmjopen-2024-086724)
Supplement: Supplementary data [file bmjopen-2024-086724supp001.pdf]

S1 Appendix: Six week Quality of life questionnaire

Date added to REDCap 6 week QoL ________________________________

6 Week QoL Questionnaire

You kindly agreed to take part in the Chapter cohort study following the birth of your baby. This research aims to understand how common complications are amongst women who have experienced childbirth acquired perineal trauma. To do this, we would like to collect some information from you at 6 weeks, 6 months and 12 months following the birth of your baby.

We have used three words frequently in the questionnaire - perineum, stitches and tear. Perineum is the part of your body between the opening of your vagina and your back passage. Stitches and tear refer to the cuts or tears in your perineum or vaginal area (either made by the midwife/doctor or happening on their own) during the birth of your baby.The perineum connects with the muscles in the lower pelvis to form the pelvic floor. Your pelvic floor helps to control your bladder, bowel and sexual function.

We expect the questionnaire will take you approximately 20 minutes to complete. It is divided into 4 sections:

Section 1: This section asks in detail about the healing of your stitches/tear and how satisfied you felt about the support you received from healthcare professionals in relation to the healing of your perineum. It asks whether you have had an infection or any complications and whether you have needed to be readmitted to hospital for treatment.

Section 2: This section explores if any symptoms related to the healing of your stitches/tear may have impacted the experience of feeding and caring for your baby (and older children, if applicable).

Section 3: This section will focus on specific questions relating to whether there have been any changes to your pelvic floor and how this impacts your bladder and bowel function. It also asks how these changes influence your day-to-day life.

Section 4: The last part of the questionnaire asks several questions about your mental health and wellbeing.

Within the Chapter study we use the term woman to refer to all birthing people as we acknowledge that it is not only people who identify as women who may be affected by childbirth related perineal trauma.

Sharing your experiences with us will increase our knowledge and lead to better understanding in this area. All the information that you provide will be confidential and may help us to improve the care women receive following childbirth.

By completing this questionnaire, you are confirming that you are still happy to take part in this research. If you decide you wish to withdraw from this study or have any queries, please contact the Chapter study research team on chapter@contacts.bham.ac.uk.

If at any time you feel this questionnaire is distressing you. you can use the 'save & return later' button to leave. If you wish you can return and complete the rest of the questionnaire at a more convenient time.

Thank you again for your participation and please remember that we are more than happy for you to contact us if you need any assistance with completing the questionnaire.

Hodgetts Morton V, *et al. BMJ Open* 2024; 14:e086724. doi: 10.1136/bmjopen-2024-086724

Section 1: This section asks in detail about the healing of your stitches/tear and how satisfied you felt about the support you received from healthcare professionals in relation to the healing of your perineum. It asks whether you have had an infection or any complications and whether you have needed to be readmitted to hospital for treatment.

Have you had any problems with the healing of your stitches or tear?

◯ Yes ◯ No

Since the birth of your baby have you noticed any discharge or leakage of fluid from any part of the stitches/tear?

◯ Yes ◯ No

Was the discharge or leakage either:

◯ Clear or blood stained
◯ Yellow/ green (pus)
◯ Other,please specify

Describe discharge or leakage:

____

Please tick any of the following additional symptoms that apply to your stitches/ tear: ______

______

When did you first notice any of these symptoms?

◯ 0-2 weeks after having my baby
◯ 2-4 weeks after having my baby
◯ 4-6 weeks after having my baby
◯ Unsure

Please check you have not missed any questions above before you continue to the next section. If you do not want to answer any questions for any personal reasons, that is fine.

Did you see a health care professional for any problems connected with your stitches or tear during this time?

◯ Yes ◯ No

Please indicate which health care professional you saw in the period following the birth of your baby for any problems connected with your stitches/tear

Doctor in hospital

◯ Yes ◯ No

How satisfied you were with the support you were given in relation to the healing of your stitches/tear from the Doctor at hospital

◯ Very satisfied
◯ Satisfied
◯ Unsatisfied
◯ Very unsatisfied

Hodgetts Morton V, *et al. BMJ Open* 2024; 14:e086724. doi: 10.1136/bmjopen-2024-086724

How satisfied you were with the support you were given in relation to the healing of your stitches/tear from the Doctor at hospital

- ◯ Very satisfied
- ◯ Satisfied
- ◯ Neutral
- ◯ Unsatisfied
- ◯ Very unsatisfied

Midwife at hospital

◯ Yes ◯ No

How satisfied you were with the support you were given in relation to the healing of your stitches/tear from the Midwife

- ◯ Very satisfied
- ◯ Satisfied
- ◯ Unsatisfied
- ◯ Very unsatisfied

How satisfied you were with the support you were given in relation to the healing of your stitches/tear from the Midwife

- ◯ Very satisfied
- ◯ Satisfied
- ◯ Neutral
- ◯ Unsatisfied
- ◯ Very unsatisfied

Midwife in community

◯ Yes ◯ No

How satisfied you were with the support you were given in relation to the healing of your stitches/tear from the Midwife

- ◯ Very satisfied
- ◯ Satisfied
- ◯ Unsatisfied
- ◯ Very unsatisfied

How satisfied you were with the support you were given in relation to the healing of your stitches/tear from the Midwife

- ◯ Very satisfied
- ◯ Satisfied
- ◯ Neutral
- ◯ Unsatisfied
- ◯ Very unsatisfied

Your General Practioner (GP)

◯ Yes ◯ No

Hodgetts Morton V, *et al. BMJ Open* 2024; 14:e086724. doi: 10.1136/bmjopen-2024-086724

How satisfied you were with the support you were given in relation to the healing of your stitches/tear from the GP

◯ Very satisfied
◯ Satisfied
◯ Unsatisfied
◯ Very unsatisfied

How satisfied you were with the support you were given in relation to the healing of your stitches/tear from the GP

◯ Very satisfied
◯ Satisfied
◯ Neutral
◯ Unsatisfied
◯ Very unsatisfied

Health visitor

◯ Yes ◯ No

How satisfied you were with the support you were given in relation to the healing of your stitches/tear from the Health visitor

◯ Very satisfied
◯ Satisfied
◯ Unsatisfied
◯ Very unsatisfied

How satisfied you were with the support you were given in relation to the healing of your stitches/tear from the Health visitor

◯ Very satisfied
◯ Satisfied
◯ Neutral
◯ Unsatisfied
◯ Very unsatisfied

Physiotherapist at the hospital

◯ Yes ◯ No

How ssatisfied you were with the support you were given in relation to the healing of your stitches/tear from the Physiotherapist

◯ Very satisfied
◯ Satisfied
◯ Unsatisfied
◯ Very unsatisfied

How ssatisfied you were with the support you were given in relation to the healing of your stitches/tear from the Physiotherapist

◯ Very satisfied
◯ Satisfied
◯ Neutral
◯ Unsatisfied
◯ Very unsatisfied

Private physiotherapy

◯ Yes ◯ No

How satisfied you were with the support you were given in relation to the healing of your stitches/tear from the private Physiotherapist

◯ Very satisfied
◯ Satisfied
◯ Unsatisfied
◯ Very unsatisfied

How satisfied you were with the support you were given in relation to the healing of your stitches/tear from the private Physiotherapist

◯ Very satisfied
◯ Satisfied
◯ Neutral
◯ Unsatisfied
◯ Very unsatisfied

Other heathcare professional

◯ Yes ◯ No

Confirm which other healthcare professional you have received advice and support from:

____

How satisfied you were with the support you were given in relation to the healing of your stitches/tear from the [anode_hcw_other_name]

◯ Very satisfied
◯ Satisfied
◯ Unsatisfied
◯ Very unsatisfied

How satisfied you were with the support you were given in relation to the healing of your stitches/tear from the [anode_hcw_other_name]

◯ Very satisfied
◯ Satisfied
◯ Neutral
◯ Unsatisfied
◯ Very unsatisfied

You have indicated that you didn't seek any further assistance from a health care professional in relation to your stitches/tear. Please indicate why you did not seek assistance. You can tick more than one - please tick ALL that apply.

☐ I didn't feel I needed to as I recovered well after the birth
☐ I wasn't aware of any services that could help me
☐ I was too embarrassed to discuss my symptoms and concerns
☐ I was fearful about an intimate examination
☐ I had some concerns and knew services existed but I haven't been able to find the time to seek further assistance
☐ Other

PLease specify Other reason you did not seek assistance.

____

Please check you have not missed any questions above before you continue to the next section. If you do not want to answer any questions for any personal reasons, that is fine.

Hospital Visits

Did you have to go to a hospital clinic as an out-patient for any problems connected with your stitches/tear since the birth of your baby?

○ Yes ○ No

What was the reason you attended the hospital as an out-patient? You can tick more than one - please tick ALL that apply.

☐ Pain or soreness in addition to the discomfort experienced following the birth
☐ Redness or inflammation spreading from the edges of the stitches/tear
☐ The area around the stitches/tear felt warmer/hotter than the surrounding skin
☐ The area around the stitches/tear became swollen
☐ The edges of any part of the stitches/tear separated or gaped open
☐ Other, please specify

Confirm the other reason you had to go to out-patients

____

How many times have you had to go to out-patients

____

Please check you have not missed any questions above before you continue to the next section. If you do not want to answer any questions for any personal reasons, that is fine.

Have you been readmitted to hospital for any reason connected with your own health since the birth of your baby? (We don't need to know if you were admitted with your baby because your baby was unwell)

○ Yes ○ No

How many times have you been readmitted for any reason connected with your own health since the birth of your baby?

○ 1 ○ 2 ○ 3 ○ more than 3

Please tell us about your first readmission to hospital by providing the following details.

Name of hospital of 1st re-admission

____

What was the reason for this first readmission? You can tick more than one - please tick ALL that apply.

☐ Pain or soreness in addition to the discomfort experienced following the birth
☐ Redness or inflammation spreading from the edges of the stitches/tear
☐ The area around the stitches/tear felt warmer/hotter than the surrounding skin
☐ The area around the stitches/tear became swollen
☐ The edges of any part of the stitches/tear separated or gaped open
☐ Other, please specify

Please specify Other reason for 1st re-admission

____

How long after you gave birth to your baby were you readmitted to hospital the first time?

◯ Less than 1 week
◯ 1-2 weeks
◯ 2-3 weeks
◯ 3-4 weeks
◯ 4-5 weeks
◯ 5-6 weeks

Did you have an operation when you were readmitted the first time?

◯ Yes ◯ No

Were you in the intensive care unit when you were readmitted the first time?

◯ Yes ◯ No

How many nights did you stay in hospital during this first readmission?

____

Please tell us about your second readmission to hospital by providing the following details.

Name of hospital of 2nd re-admission

____

What was the reason for this second readmission? You can tick more than one - please tick ALL that apply.

☐ Pain or soreness in addition to the discomfort experienced following the birth
☐ Redness or inflammation spreading from the edges of the stitches/tear
☐ The area around the stitches/tear felt warmer/hotter than the surrounding skin
☐ The area around the stitches/tear became swollen
☐ The edges of any part of the stitches/tear separated or gaped open
☐ Other, please specify

Please specify Other reason for 2nd re-admission

____

How long after you gave birth to your baby were you readmitted to hospital the second time?

◯ Less than 1 week
◯ 1-2 weeks
◯ 2-3 weeks
◯ 3-4 weeks
◯ 4-5 weeks
◯ 5-6 weeks

Did you have an operation when you were readmitted the second time?

◯ Yes ◯ No

Were you in the intensive care unit when you were readmitted the first time?

◯ Yes ◯ No

How many nights did you stay in hospital during this second readmission?

____

Please tell us about your third readmission to hospital by providing the following details.

Name of hospital of 3rd re-admission

____

What was the reason for this third readmission? You can tick more than one - please tick ALL that apply.

☐ Pain or soreness in addition to the discomfort experienced following the birth
☐ Redness or inflammation spreading from the edges of the stitches/tear
☐ The area around the stitches/tear felt warmer/hotter than the surrounding skin
☐ The area around the stitches/tear became swollen
☐ The edges of any part of the stitches/tear separated or gaped open
☐ Other, please specify

Please specify Other reason for 3rd re-admission

____

How long after you gave birth to your baby were you readmitted to hospital the third time?

◯ Less than 1 week
◯ 1-2 weeks
◯ 2-3 weeks
◯ 3-4 weeks
◯ 4-5 weeks
◯ 5-6 weeks

Did you have an operation when you were readmitted the third time?

◯ Yes ◯ No

Were you in the intensive care unit when you were readmitted the third time?

◯ Yes ◯ No

How many nights did you stay in hospital during this third readmission?

____

As you have been re-admitted to hospital more than 3 times please write how many ADDITIONAL nights you have stayed

____

Please check you have not missed any questions above before you continue to the next section. If you do not want to answer any questions for any personal reasons, that is fine.

Have you been prescribed any antibiotics since the birth of your baby? (if you were readmitted to hospital include any antibiotics you were given then).

◯ Yes
◯ No
◯ Unknown drug received
◯ Cannot remember if any medication was received

What were they given for?

☐ Pain or soreness in addition to the discomfort experienced following the birth
☐ Redness or inflammation spreading from the edges of the stitches/tear
☐ The area around the stitches/tear felt warmer/hotter than the surrounding skin
☐ The area around the stitches/tear became swollen
☐ The edges of any part of the stitches/tear separated or gaped open
☐ Other, please specify

Other reason drugs were given

____

Who prescribed them?- please tick ALL that apply.

☐ GP
☐ Doctor in maternity unit
☐ Doctor in emergency department
☐ Other

Other prescriber, please specify

____

Were they prescribed more than 24 hours after the birth of your baby?

◯ Yes ◯ No

Please check you have not missed any questions above before you continue to the next section. If you do not want to answer any questions for any personal reasons, that is fine.

Has your doctor taken any bloods or swabs to send to the lab to check for infection

◯ Yes
◯ No
◯ Don't know

Have you been told you had an infection in your perineum

◯ Yes
◯ No
◯ Unsure

Please tick all signs and symptoms you experienced:

☐ Fever >38 degrees
☐ Perineal pain
☐ Smelly discharge
☐ Redness around the stiches/tear
☐ Swelling
☐ Other

Other signs and symptoms you experienced:

____

Please check you have not missed any questions above before you continue to the next section. If you do not want to answer any questions for any personal reasons, that is fine.

In the past week, have you experienced any pain or unpleasant feeling in your perineum? (the area between your vagina and back passage)

◯ Yes ◯ No

Would you describe the strength of the pain or unpleasant feeling as?

◯ Mild
◯ Moderate
◯ Severe

Is the pain or unpleasant feeling there?

◯ Some of the time
◯ Most of the time
◯ All of the time

In the past week have you needed any tablets to relieve the pain or discomfort in your perineum?

◯ Yes ◯ No

Please check you have not missed any questions above before you continue to the next section. If you do not want to answer any questions for any personal reasons, that is fine.

Hodgetts Morton V, *et al. BMJ Open* 2024; 14:e086724. doi: 10.1136/bmjopen-2024-086724

How well do you feel that your perineum has healed? .   (Please tick one box that is the most appropriate statement for the way you feel)

◯ I feel that my perineum has healed well
◯ I feel that my perineum has healed satisfactorily
◯ I feel that my perineum has healed poorly
◯ I feel that my perineum has healed very poorly

Please check you have not missed any questions above before you continue to the next section. If you do not want to answer any questions for any personal reasons, that is fine.

Section 2: This section explores if any symptoms related to the healing of your stitches/tear may have impacted the experience of feeding and caring for your baby (and older children, if applicable).

Have you breastfed your baby at any time since he/she was born

◯ Yes ◯ No

Are you still breastfeeding your baby

◯ Yes ◯ No

If you are no longer breastfeeding your baby: how old was your baby when you stopped breastfeeding? Please specify to the closest week breastfeeding stopped.

◯ Less than 1 week
◯ Between 1-2 weeks
◯ Between 2-3 weeks
◯ Between 3-4 weeks
◯ Between 4-5 weeks
◯ Between 5-6 weeks

Why did you stop breastfeeding You can tick more than one - please tick ALL that apply.

☐ Painful nipples
☐ Engorgement
☐ Blocked milk duct
☐ Thrush
☐ Mastitis
☐ Breast abscess
☐ Not enough milk
☐ Perineum was too uncomfortable or painful whilst feeding my baby
☐ Other

Other reason stopped

____

Has your perineum been too uncomfortable or painful for you to feed your baby

◯ Yes ◯ No

Have problems or worries about your perineum caused you to not be able to care for your baby in the way you'd like to during this time? (Care includes washing/ feeding/ playing/ holding your baby).

◯ Yes
◯ No
◯ I have not had any problems or worries about my perineum

How often do these problems or worries about your perineum interfere with you caring for your baby:

◯ A lot of the time (for example, more than once a day)
◯ Some of the time (for example, at least once a day)
◯ Not very often

Do you feel problems or worries about your perineum have affected bonding with your baby during this time?

◯ Yes
◯ No
◯ I have not had any problems or worries about my perineum

Have problems or worries about your perineum caused you to not be able to care for your older children in the way you'd like?

◯ Yes
◯ No
◯ I don't have any older children

How often do these problems or worries about your perineum interfere with you caring for your older child?

◯ A lot of the time (for example, more than once a day)
◯ Some of the time (for example, at least once a day)
◯ Not very often

Please check you have not missed any questions above before you continue to the next section. If you do not want to answer any questions for any personal reasons, that is fine.

Section 3: This section will focus on specific questions relating to whether there have been any changes to your pelvic floor and how this impacts your bladder and bowel function. It also asks how these changes influence your relationships and day-to-day life

Please consider your experiences during the last 4 weeks for the following.

Have you had any problems with controlling your bowels? (This includes passing of wind (flatus) and stool).

◯ Yes ◯ No

Do you have difficulty controlling wind (flatus)?

◯ Never
◯ Rarely (for example less than once in the past 4 weeks)
◯ Sometimes (for example less than once a week, but once or more in the past 4 weeks)
◯ Often or usually (for example less than once a day but once a week or more)
◯ Always (for example once or more per day or whenever you have a bowel movement)

Do you leak, have accidents or lose control with solid stool

◯ Never
◯ Rarely i.e. less than once in the past four weeks
◯ Sometimes i.e. less than once a week, but once or more in the past four weeks
◯ Often or usually i.e. less than once a day but once a week or more
◯ Always i.e. once or more per day or whenever you have a bowel movement

Do you leak, have accidents or lose control with liquid stool

◯ Never
◯ Rarely i.e. less than once in the past four weeks
◯ Sometimes i.e. less than once a week, but once or more in the past four weeks
◯ Often or usually i.e. less than once a day but once a week or more
◯ Always i.e. once or more per day or whenever you have a bowel movement

Do you leak stool if you don't get to the toilet in time?

◯ Never
◯ Rarely i.e. less than once in the past four weeks
◯ Sometimes i.e. less than once a week, but once or more in the past four weeks
◯ Often or usually i.e. less than once a day but once a week or more
◯ Always i.e. once or more per day or whenever you have a bowel movement

Does stool leak so that you have to change your underwear

◯ Never
◯ Rarely i.e. less than once in the past four weeks
◯ Sometimes i.e. less than once a week, but once or more in the past four weeks
◯ Often or usually i.e. less than once a day but once a week or more
◯ Always i.e. once or more per day or whenever you have a bowel movement

Does bowel or stool leakage cause you to alter your lifestyle

◯ Never
◯ Rarely i.e. less than once in the past four weeks
◯ Sometimes i.e. less than once a week, but once or more in the past four weeks
◯ Often or usually i.e. less than once a day but once a week or more
◯ Always i.e. once or more per day or whenever you have a bowel movement

Please check you have not missed any questions above before you continue to the next section. If you do not want to answer any questions for any personal reasons, that is fine.

Please consider your experiences during the last 4 weeks for the following.

Have you had any problems with controlling your bladder?

◯ Yes ◯ No

Do you experience, and if so, how much are you bothered by:

Urine leakage related to the feeling of urgency

◯ Not at all
◯ Slightly
◯ Moderately
◯ Greatly

Urine leakage related to physical activity, coughing or sneezing

◯ Not at all
◯ Slightly
◯ Moderately
◯ Greatly

Small amounts of urine leakage (drops)

◯ Not at all
◯ Slightly
◯ Moderately
◯ Greatly

How often do you experience urine leakage

◯ Never
◯ Less than once a month
◯ A few times a month
◯ A few times a week
◯ Every day and/or night

How much urine do you lose each time

◯ None
◯ Drops
◯ Small splashes
◯ More

Please check you have not missed any questions above before you continue to the next section. If you do not want to answer any questions for any personal reasons, that is fine.

Under each heading, please select the ONE box that best describes your health TODAY

MOBILITY

◯ I have no problems in walking about
◯ I have slight problems in walking about
◯ I have moderate problems in walking about
◯ I have severe problems in walking about
◯ I am unable to walk about

SELF-CARE

◯ have no problems washing or dressing myself
◯ I have slight problems washing or dressing myself
◯ I have moderate problems washing or dressing myself
◯ I have severe problems washing or dressing myself
◯ I am unable to wash or dress myself

USUAL ACTIVITIES (e.g. work, study, housework, family or leisure activities)

◯ I have no problems doing my usual activities
◯ I have slight problems doing my usual activities
◯ I have moderate problems doing my usual activities
◯ I have severe problems doing my usual activities
◯ I am unable to do my usual activities

PAIN / DISCOMFORT

◯ I have no pain or discomfort
◯ I have slight pain or discomfort
◯ I have moderate pain or discomfort
◯ I have severe pain or discomfort
◯ I have extreme pain or discomfor

ANXIETY / DEPRESSION

◯ I am not anxious or depressed
◯ I am slightly anxious or depressed
◯ I am moderately anxious or depressed
◯ I am severely anxious or depressed
◯ I am extremely anxious or depressed

We would like to know how good or bad your health is TODAY. This scale is numbered from 0 to 100.
100 means the best health you can imagine.
0 means the worst health you can imagine.
Please click on the scale to indicate how your health is TODAY.

0 - The worst health you can imagine	50	100 - The best health you can imagine

(Place a mark on the scale above)

Please check you have not missed any questions above before you continue to the next section. If you do not want to answer any questions for any personal reasons, that is fine.

Section 4: The last part of the questionnaire asks several questions about your mental health and wellbeing.

While it is common to feel a little bit low after having a baby, we would like you to know that there are people who can provide support or information if you are struggling to cope with this.

Getting the right help from a health professional early on leads to a faster recovery. Please speak to your GP or health visitor if there is anything that is troubling you and they will be able to support you. You may also find some of the resources at the end of this questionnaire helpful too.

In the below questions please select the answer that comes closest to how you have felt IN THE PAST 7 DAYS, not just how you feel today.

I have been able to laugh and see the funny side of things

◯ As much as I always could
◯ Not quite so much now
◯ Definitely not so much now
◯ Not at all

I have looked forward with enjoyment to things

◯ As much as I ever did
◯ Rather less than I used to
◯ Definitely less than I used to
◯ Hardly at all

I have blamed myself unnecessarily when things went wrong

◯ Yes, most of the time
◯ Yes, some of the time
◯ Not very often
◯ No, never

I have been anxious or worried for no good reason

◯ No, not at all
◯ Hardly ever
◯ Yes, sometimes
◯ Yes, very often

I have felt scared or panicky for no very good reason

◯ Yes, quite a lot
◯ Yes, sometimes
◯ No, not much
◯ No, not at all

Things have been getting on top of me

◯ Yes, most of the time I haven't been able to cope at all
◯ Yes, sometimes I haven't been coping as well as usual
◯ No, most of the time I have coped quite well
◯ No, I have been coping as well as ever

Please check you have not missed any questions above before you continue to the next section. If you do not want to answer any questions for any personal reasons, that is fine.

Supplemental material BMJ Publishing Group Limited (BMJ) disclaims all liability and responsibility arising from any reliance placed on this supplemental material which has been supplied by the author(s) BMJ Open

This following questions asks about your experience during the birth of your most recent baby. It asks about potential traumatic events during (or immediately after) the labour and birth, and whether you are experiencing symptoms that are reported by some women after birth and how often you have experienced these in the last week. Please tick the responses closest to your experience.

Recurrent unwanted memories of the birth (or parts of the birth) that you can't control

◯ Not at all
◯ Once
◯ 2-4 times
◯ 5 or more times

Bad dreams or nightmares about the birth (or related to the birth)

◯ Not at all
◯ Once
◯ 2-4 times
◯ 5 or more times

Flashbacks to the birth and/or reliving the experience

◯ Not at all
◯ Once
◯ 2-4 times
◯ 5 or more times

Getting upset when reminded of the birth

◯ Not at all
◯ Once
◯ 2-4 times
◯ 5 or more times

Feeling tense or anxious when reminded of the birth

◯ Not at all
◯ Once
◯ 2-4 times
◯ 5 or more times

Trying to avoid things that remind me of the birth (for example, people, places, TV programmes)

◯ Not at all
◯ Once
◯ 2-4 times
◯ 5 or more times

Not able to remember details of the birth

◯ Not at all
◯ Once
◯ 2-4 times
◯ 5 or more times

Hodgetts Morton V, *et al. BMJ Open* 2024; 14:e086724. doi: 10.1136/bmjopen-2024-086724

Blaming myself or others for what happened during the birth

◯ Not at all
◯ Once
◯ 2-4 times
◯ 5 or more times

Feeling strong negative emotions about the birth (for example, fear, anger, shame)

◯ Not at all
◯ Once
◯ 2-4 times
◯ 5 or more times

When did these symptoms start?

◯ Before the birth
◯ In the first 6 weeks after the birth

Do these symptoms cause you a lot of distress?

◯ Yes
◯ No
◯ Sometimes

How long have these symptoms lasted?

◯ Less than 1 month
◯ 1 to 2 months

Do they prevent you doing things you usually do (for example, socialising, daily activities?)

◯ Yes
◯ No
◯ Sometimes

Could any of these symptoms be due to medication, alcohol, drugs or physical illness?

◯ Yes
◯ No
◯ Sometimes

Please check you have not missed any questions above before you continue to the next section. If you do not want to answer any questions for any personal reasons, that is fine.

You may have found answering some of these questions distressing. The research team can contact you by telephone to offer support or you can contact your GP. Please indicate below whether you would like the research team to contact you.

◯ I would like to receive a telephone call to offer support.
◯ I do not wish to receive a support call.

Additonal Information

Is there anything else you would like to tell us about the healing of your stitches/tear or how you are feeling now? Please feel free to make any comments important to you in this space.

Thank you very much for taking the time to complete this questionnaire. We will be contacting you to ask you to complete the next questionnaire approximately 6 months following the birth of your baby. If you would like to speak to the Chapter research team about any aspect of this study, please contact the team by email chapter@contacts.bham.ac.uk.

Should you need further support or information at any point please speak to your GP or health visitor. You may find the following resources helpful: www.nhs.uk - to find information and advice on health conditions, symptoms and how to get help www.masic.org.uk - a charity which supports women who have sustained severe perineal trauma during childbirth www.mind.org.uk - a charity that provides advice and support to empower anyone experiencing a mental health problem www.pandasfoundation.org.uk - a charity which provides support for every parent who is struggling with their perinatal mental health
