# Supplementary figures and images for: Childbirth Acquired Perineal Trauma study (CHAPTER): a UK prospective cohort study protocol
Source: BMJ Open. 2024 May 24;14(5):e086724. doi: 10.1136/bmjopen-2024-086724 (PMC11129024; doi:10.1136/bmjopen-2024-086724)

S2 Appendix: Distress Pathway

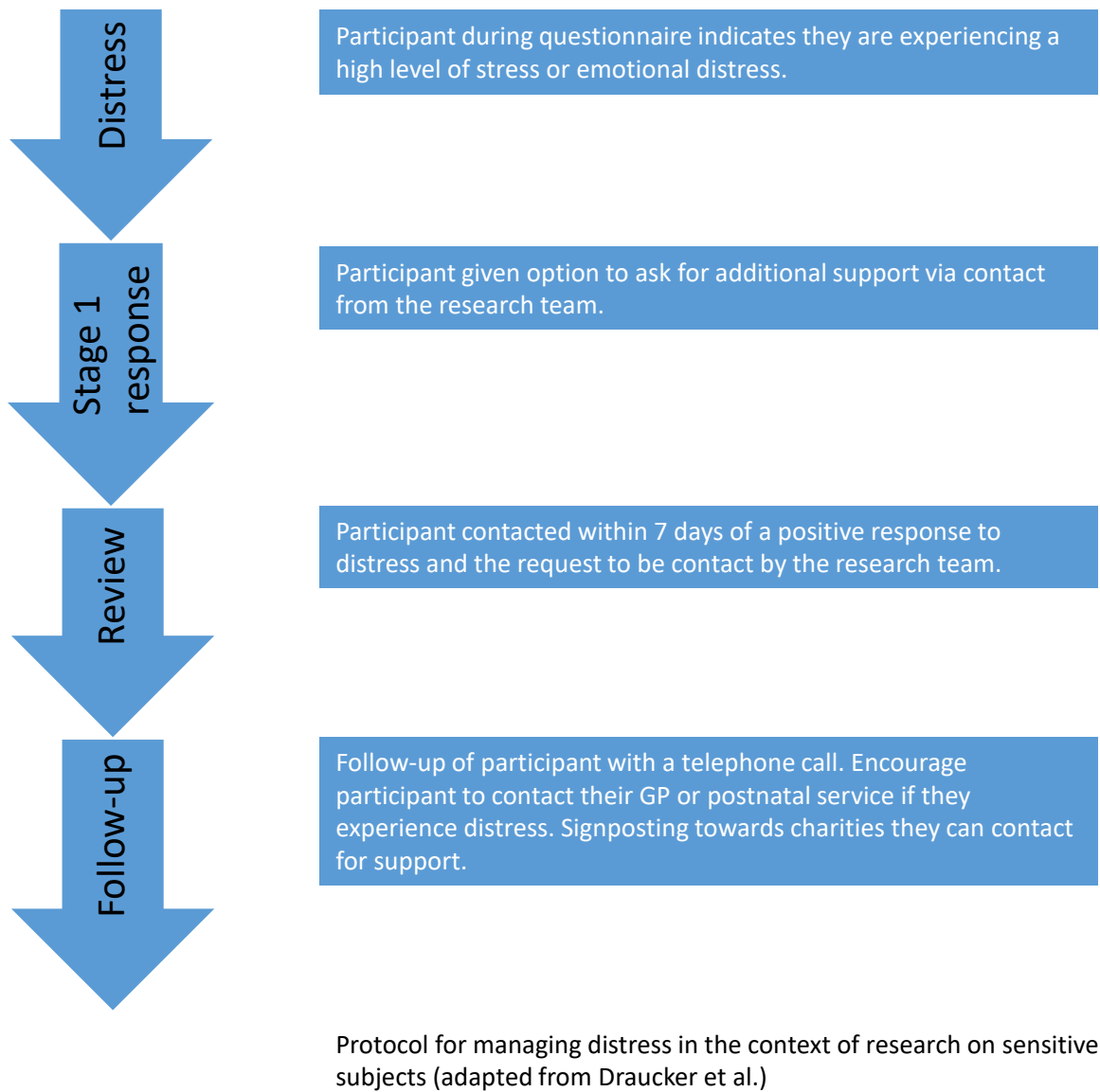

Supplement: Supplementary data [file bmjopen-2024-086724supp002.pdf]
