# Supplementary material for: Childbirth Acquired Perineal Trauma study (CHAPTER): a UK prospective cohort study protocol
Source: BMJ Open. 2024 May 24;14(5):e086724. doi: 10.1136/bmjopen-2024-086724 (PMC11129024; doi:10.1136/bmjopen-2024-086724)
Supplement: Author's manuscript [file bmjopen-2024-086724.draft_revisions.pdf]

BMJ Open

BMJ Open is committed to open peer review. As part of this commitment we make the peer review history of every article we publish publicly available.

When an article is published we post the peer reviewers' comments and the authors' responses online. We also post the versions of the paper that were used during peer review. These are the versions that the peer review comments apply to.

The versions of the paper that follow are the versions that were submitted during the peer review process. They are not the versions of record or the final published versions. They should not be cited or distributed as the published version of this manuscript.

BMJ Open is an open access journal and the full, final, typeset and author-corrected version of record of the manuscript is available on our site with no access controls, subscription charges or pay-per-view fees (http://bmjopen.bmj.com).

If you have any questions on BMJ Open's open peer review process please email info.bmjopen@bmj.com

BMJ Open

CHILDBIRTH ACQUIRED PERINEAL TRAUMA STUDY (CHAPTER): A PROSPECTIVE COHORT STUDY PROTOCOL

Journal:	*BMJ Open*
Manuscript ID	bmjopen-2024-086724
Article Type:	Protocol
Date Submitted by the Author:	21-Mar-2024
Complete List of Authors:	Hodgetts Morton, Victoria ; Birmingham Women's Hospital; University of Birmingham Institute of Applied Health Research<br>Man, Rebecca; University of Birmingham Institute of Applied Health Research<br>Perry, Rita; University of Birmingham Institute of Applied Health Research<br>Hughes, Terry; University of Birmingham Institute of Applied Health Research<br>Tohill, Susan; University of Birmingham Institute of Applied Health Research<br>MacArthur, Christine; University of Birmingham Institute of Applied Health Research, Public Health<br>Magill, Laura ; University of Birmingham Institute of Applied Health Research<br>Morris, R. K.; Birmingham Women's Hospital; University of Birmingham Institute of Applied Health Research<br>Group, CHAPTER ; The CHAPTER Collaborative
Keywords:	Postpartum Period, Natural Childbirth, Pregnant Women, Postpartum Women < Postpartum Period

SCHOLARONE™
Manuscripts

CHILDBIRTH ACQUIRED PERINEAL TRAUMA STUDY (CHAPTER): A PROSPECTIVE COHORT STUDY PROTOCOL

Victoria Hodgetts Morton[1,2], Rebecca Man[1]*, Rita Perry[1], Terry Hughes[1], Sue Tohill[1], Christine MacArthur[1], Laura Magill[1], R Katie Morris[1,2] on behalf of the CHAPTER group**

1. Institute of Applied Health Research, University of Birmingham, Birmingham, United Kingdom

2. Birmingham Women's and Children's NHS Foundation Trust, Birmingham, United Kingdom

*Corresponding author

Email: r.man@bham.ac.uk

Keywords: postpartum period, natural childbirth, pregnant women, postpartum women

For peer review only - http://bmjopen.bmj.com/site/about/guidelines.xhtml

CHILDBIRTH ACQUIRED PERINEAL TRAUMA STUDY (CHAPTER): A PROSPECTIVE COHORT STUDY PROTOCOL

Abstract

Introduction

Childbirth related perineal trauma (CRPT) is the commonest complication of childbirth affecting 80% of women overall after vaginal birth. There remains a lack of comprehensive evidence relating to the prevalence of subsequent health problems. Current evidence is related to short-term outcomes, for example pain, but there is less known about longer term outcomes such as infection, wound dehiscence, pelvic floor function and psychological outcomes. This is a protocol for a cohort study assessing outcomes of women after CRPT.

Methods and analysis

A multi-centre, prospective UK cohort study aiming to include 1000 women. All women who have sustained CRPT will be eligible for inclusion and will be followed-up for 12 months after childbirth. The primary outcome will be perineal infection at six weeks post-birth. Secondary outcomes will include antibiotic use for perineal infection, wound breakdown, use of analgesia, requirement for admission or surgical intervention, urinary and faecal incontinence, anxiety and depressive symptoms, sexual function and impact on daily activities. Outcomes will be measured at six weeks, six months and 12 months postpartum, with some outcomes being measured at all time-points and others at selected most appropriate time-points only. Outcome data will be obtained from review of clinical notes and from patient questionnaires. Simple descriptive statistics will be used to summarize characteristics and outcomes, with categorical variables expressed as percentages and continuous variables as mean averages, alongside the corresponding standard deviation.

For peer review only - http://bmjopen.bmj.com/site/about/guidelines.xhtml

Ethics and dissemination

Ethical approval has been granted by the Research Ethics Council with reference 23/WA/0169. Data collected from the Childbirth Acquired Perineal Trauma (CHAPTER) cohort study will highlight the prevalence and type of complications after CRPT and which women are more at risk. After the conclusion of this study, findings will be used to work with governmental organisations and Royal Colleges to target resources and ultimately improve care.

Article Summary

Strengths and limitations

- This study aims to incorporate a diverse range of women with childbirth related perineal trauma (CRPT) from across the UK, with the goal of producing representative and up-to-date information on complications after CRPT.
- Follow-up questionnaires are comprehensive and holistic with a range of potential physical and psychological complications after CRPT being assessed.
- Follow-up for the year after childbirth is integral to this study and we will need to consider the potential for non-response to the questionnaires, particularly at the later data collection points.
- Questionnaires will need to be completed and returned within the specified time-frame in relation to the follow-up points. Where responses occur outside these time windows we will need to consider the impact of this on response.

For peer review only

Introduction

In the UK every year, 80% of women who have a vaginal birth (approximately 450,000 women), experience childbirth related perineal trauma (CRPT) [1, 2]. CRPT affects 91% of women in a first birth and 70% in successive births and is therefore the most common complication after childbirth [2]. Despite CRPT affecting the vast majority of women who give birth, the prevalence and severity of resulting health complications remains unclear. There is some evidence relating to initial outcomes, for example pain,

For peer review only - http://bmjopen.bmj.com/site/about/guidelines.xhtml

but less is known about longer-term complications, including; wound dehiscence, infection, psychological or social issues, sexual dysfunction and incontinence [3-6].

Complications from CRPT can be multi-faceted and long-lasting, with some women suffering wound related complications for many years and ultimately requiring complex reconstructive surgery, or developing long-term psychological problems [7, 8]. There is currently very little research exploring the prevalence of severe CRPT, its risk factors, management or its short or long-term complications [9]. Information to tell us how serious these complications are or how often they occur, is not routinely collected. There is very little research to indicate which women might be more likely to suffer complications from CRPT or experience longer-term health problems [3-6]. The Childbirth Acquired Perineal Trauma (CHAPTER) cohort study is designed to address this substantial evidence gap.

This multicentre, prospective, UK-based cohort study will follow a diverse group of UK women who have sustained CRPT. We will determine the current assessment and management of CRPT immediately after it is sustained, in addition to the prevalence and type of short- and long-term complications in women who have CRPT. Risk factors for developing CRPT complications and current practice of assessment and management of CRPT complications will be ascertained.

Methods and analysis

Objectives

• To describe the current assessment and management of CRPT at the time it is sustained

• To determine the prevalence of short- and long-term complications or health problems in women who have CRPT

• To describe the management of subsequent complications in women with CRPT

• To describe the risk factors associated with the development of CRPT associated complications or longer-term sequalae

For peer review only - http://bmjopen.bmj.com/site/about/guidelines.xhtml

Study design

A UK multi-centre prospective cohort study

Study setting

CHAPTER is open to any type of unit in the UK which offers maternity care. This includes obstetric-led, alongside midwifery units and freestanding midwifery units.

Sample size calculations

We will aim to recruit a minimum of 1000 women. 300 participants will allow us to determine a CRPT complication prevalence rate of 10% with a 95% confidence interval (CI) of 6.8%-14.0%. A sample size of 900 will allow rarer outcomes to be estimated with precision, for example a 95% CI for an estimate of 5% would range from 3.7% to 6.6%. We anticipate the sample will include approximately 450 women in the minor trauma group and 450 women in the second, third and fourth degree tear group. We have in excess of 99% power to detect the difference in pain relief previously seen and with the anticipated sample we would be able to detect a difference of less than 10 percentage points between groups (25% vs 35%).

Approximately 70% of women have a vaginal delivery (55-60% unassisted; 10-15% assisted) and there are 73,889 live births per six week period, thus 51,722 with a vaginal delivery of which 39,723 will sustain non-obstetric anal sphincter injury (non-OASI) CPRT and 2,068 OASI CRPT [1, 10]. Based on 20 maternity sites participating (n=9,930) and 80% of those being suitable to be approached (eligible, baby or mother not too unwell to be approached – 7,944), if 50% of those eligible are successfully approached with 50% consenting (1,986) and 50% completing all follow-up gives a potential sample of n=993 for all outcomes. For those outcomes that can be determined by routine data collection this gives a potential sample of n=1,986 [11, 12].

For peer review only - http://bmjopen.bmj.com/site/about/guidelines.xhtml

Inclusion criteria

To include women who fit any of the following criteria:

- women (≥ 16 yrs) who have given birth either spontaneously or operatively and sustained CRPT (including OASI) who are able and willing to give informed consent
    - to include women who have failed instrumental delivery who ultimately give birth via caesarean section provided they have sustained CRPT
    - to include women with multiple births who give birth vaginally and via caesarean section provided they have sustained CRPT

Exclusion criteria

None

Outcomes to be measured

Primary outcome: perineal infection within six weeks post-birth

Secondary outcomes:

Clinical outcomes from medical records check

- Antibiotic use for perineal infection

- Wound breakdown

- Urinary and faecal incontinence

- Readmission or triage visit for CRPT-related complications

- Requiring referral or review within a specialist perineal clinic

- Requiring minor or major corrective perineal surgery

Patient-reported outcomes as obtained from questionnaires

At six-weeks, six and 12 months post-partum:

- Urinary incontinence via the Revised Urinary Incontinence Scale (RUIS) [13]

For peer review only - http://bmjopen.bmj.com/site/about/guidelines.xhtml

- Faecal incontinence via the Revised Faecal Incontinence Scale (RFIS) [14]
- Anxiety and depression via the Edinburgh Postnatal Depression Scale [15]
- General health related quality of life using the EQ-5D-5L [16]
- Post-traumatic stress disorder (PTSD) via selected domains from the City Birth Trauma Scale [17]
- Measures of pain
- Readmissions

At six and 12-months post-partum only:

- Sexual function via the Bespoke CHAPTER Questionnaire
- Physical activity via the Pelvic Floor Impact Questionnaire [18]
- Pelvic organ prolapse symptoms via selected domains from the Revised Postpartum Pelvic Floor and Birth Questionnaire [19]
- Maternal satisfaction
- Ability to feed baby according to mother's preference
- Ability to care for baby
- Social isolation
- Ability to care for older children
- Impact on seeking healthcare
- Future pregnancy and subsequent mode of birth preference

Identification of participants and recruitment

Potentially eligible participants will be identified by members of the clinical care or research team at the relevant site. This will include midwives and obstetricians (consultants and trainees), research midwives and nurses in maternity units in secondary and tertiary care NHS Trusts. All participants screened for inclusion in the CHAPTER study will be recorded on the online CHAPTER screening log. No identifiable information will be included on the screening log. This log will be prospectively maintained at each site using either the hospital birth register or the postnatal ward-specific list of

For peer review only - http://bmjopen.bmj.com/site/about/guidelines.xhtml

births. The CHAPTER Screening Log will be held on the Research Electronic Data Capture (REDCap) system.

There will be a maximum of three defined 14-day data collection periods. We shall review demographics and type of CRPT after each 14-day data collection period to ensure that we are achieving representation related to non-OASI and our aim for inclusivity. This will allow us to purposively recruit in subsequent rounds if required.

Consent process

Within the CHAPTER Cohort Study, two different methods can be used to obtain informed consent for the study:

- In-person written informed consent
- Remote consent

Irrespective of the type of consent to be used, the first approach to potential participants for possible inclusion in the study must be made by a member of the clinical care team at site (e.g. midwife or obstetrician).

The initial approach to potential participants will, in most cases, take place while women are still an inpatient and can be in one of two settings:

- On the labour ward/birthing unit for those women anticipating an early discharge but at least six hours after birth.
- On the post-natal ward.
- For those women who have a home birth or who are discharged before they have been approached for inclusion, they will be asked to participate when they are seen by a community midwife usually at day one post-partum.

For peer review only - http://bmjopen.bmj.com/site/about/guidelines.xhtml

Women will be provided with background information about the study, including the premise and what their participation would involve. The women will have an opportunity to ask questions and a written CHAPTER study patient information sheet (PIS) will be provided as an adjunct to this conversation.

Those for whom English is not their first spoken language will also be supported by standard Trust interpretation services, for example Trust interpreters or Language Line (a UK language translation service agency that provides interpreting and translation services in over 200 languages, including British Sign Language). Women who understand spoken English will be able to participate in the study. They will be contacted via telephone and asked to complete the follow-up questionnaires over the telephone with a translator should they choose to. A record of the discussion with the women will be kept in their medical notes.

Remote consent process

Remote documented consent is an option within the CHAPTER Study. Remote consent may be undertaken if in-person consent is not feasible, for example in areas where participants are likely to have a homebirth due to the geographical area. In these instances, the woman will be provided with the PIS and consent form by the community midwife or by post. The informed consent discussions will proceed as detailed, but by telephone or video-conference and the details will be recorded in the patient's medical notes.

There are then two options which may be used to document informed consent. The choice of which method is the decision of the research team at site:

- Scanned/ photograph of consent form:

The patient will be asked to initial the boxes on the consent form, and sign and date the form whilst on the call. If the consent form was sent to the patient by post the patient will be asked to take a photo or scan of the signed form, and email it to the site staff, who will print it off and counter-sign. If the consent form was sent to the patient electronically, and they have access to a printer, they can print a

For peer review only - http://bmjopen.bmj.com/site/about/guidelines.xhtml

For peer review only

hard copy, take a photo or scan of the signed form, and email it to the site staff, who will print it off and counter-sign. The original signed and counter-signed consent form will be placed in the Investigator Site File (ISF), a copy will be sent to the participant (electronically or by post), and a copy will be filed in the patient's medical notes.

-Postal consent:

The patient will be asked to initial the boxes on the consent form, and sign and date the form whilst on the call. The patient should then post the signed consent form to the site staff, who will counter-sign it upon receipt. The original signed and counter-signed consent form will be placed in the ISF, a copy will be posted to the participant, and a copy will be filed in the patient's medical notes.

Withdrawal of consent

Participants will be made aware from the beginning of their involvement in the study that they can freely withdraw at any time. Continued consent will be assumed by the completion and return of the questionnaires at each time point. Participants will be given details on how to withdraw from the study at any point in the PIS. Reasons for withdrawal must be recorded on the CHAPTER REDCap database.

If a participant decides that they do not wish to continue their participation in the CHAPTER Study, they may withdraw in one of two ways:

1) No study related follow-up: The participant does not wish to complete patient-reported study-specific follow-up (i.e. patient reported outcomes and quality of life measures at six weeks, six- and 12-months postpartum) but is willing for routinely collected data to be used for inclusion in the study.

2) No further data collection: The participant is not willing to be followed up in any way for the purposes of the study AND does not wish for any further data to be collected (i.e., only data collected prior to withdrawal can be used in the study analysis).

For peer review only - http://bmjopen.bmj.com/site/about/guidelines.xhtml

If a participant withdraws from the CHAPTER Study, their study record on the CHAPTER REDCap database will be closed and no further data will be collected, as per the participant's request. All data collected until that time-point, will be retained and included in the analysis.

Participant Registration

Registration will be via a secure online system hosted by the University of Birmingham Centre for Prospective and Observational Studies (BiCOPS); www.CHAPTER-bicops.bham.ac.uk. Unique log-in usernames will be provided to those who wish to use the online system and who have been delegated the role of registering participants into the study.

After eligibility for registration has been confirmed and informed consent has been taken and documented, the participant can be registered to the study using the CHAPTER online system. During the online registration process, eligibility must be confirmed and participant contact details for follow-up must be entered. A Registration Number will be given once all questions have been answered. Following registration, a confirmatory e-mail will be sent to the responsible clinician, midwife and local Principle Investigator (PI). The local research team should add the participant to the CHAPTER participant recruitment and identification log which links participants with their CHAPTER study number. PIs must maintain this document securely and it must not be submitted to the study office. The CHAPTER participant recruitment and identification log should be held in strict confidence.

Once the patient has consented for entry into CHAPTER, their GP should be notified that they are in the CHAPTER study, using the CHAPTER study GP Letter. No other parties outside of the study team will be informed of the participant's entry into the study.

Baseline data collection

After the woman has consented and they are registered on to the CHAPTER study, baseline information will be collected (outline of information collected in table 1). Information will be collected

For peer review only - http://bmjopen.bmj.com/site/about/guidelines.xhtml

from the maternity record related to demographics (e.g. age, ethnicity), maternal characteristics (e.g. body mass index, obstetric history, medical conditions) and delivery characteristics (e.g. mode of delivery, CRPT type and details of repair). There are no additional study-specific follow-up hospital visits once the participant is entered into the CHAPTER study.

Table 1: A summary of baseline study participant characteristics

Category	Baseline data item
Demographic information	Age
	Ethnicity
	Geographical location
Maternal characteristics	Booking weight
	Body mass index
	Comprehensive obstetric history including previous tear details
	Medical conditions pre-existing or during pregnancy
	Smoking status
	Female genital mutilation status, type and deinfibulation
Labour and birth details	Onset of labour: spontaneous or induced
	Length of second stage
	Singleton/multiples birthed
	Mode and setting of delivery
	Presentation and position at birth
	Birth outcome: livebirth/stillbirth
	Episiotomy
	Hands on/poised/off perineum at crowning
	Maternal position
	Shoulder dystocia and management if occurred
	Blood loss
	Analgesia used in labour
	Presence of chorioamnionitis
	Use of antibiotics in labour or immediately postpartum and indication
	Baby details: gestation, sex, weight and measurements
Tear details, repair details	Grade and role of clinician who inspected perineum after birth
	Classification of tear: first degree tear, second degree tear, third degree tear, fourth degree tear, episiotomy, other lacerations
	Repair performed: method, suture material, by whom, in which setting
	Analgesia during and after repair
	Examinations performed before/after repair (vaginal examination/per rectal examination) and outcome
	Did baby stay with mother during repair?
	Was follow-up of tear arranged?

Data collection timepoints

All follow-up will be conducted remotely. At the time of study entry, participants will be asked to specify their preferred method of contact. Participants will be contacted at the follow-up timepoints, using their

For peer review only - http://bmjopen.bmj.com/site/about/guidelines.xhtml

preferred method of communication where possible to request completion of the CHAPTER study questionnaire (S1 Appendix— six-week questionnaire). There are three follow-up timepoints at six weeks, six months and 12 months postpartum.

At six weeks postpartum: A clinical note review of electronic secondary care records will be undertaken. This combined with the questionnaire data at this time-point, will be used to determine whether the primary outcome of perineal infection within six weeks post-partum has been met.

At all time-points, participants will be asked to complete questionnaire items relating to: urinary incontinence, flatus incontinence, faecal incontinence, anxiety and depressive symptoms, general health related quality of life, PTSD symptoms (please see outcomes section for specific questionnaires used).

At six and 12 months postpartum only: participants will be asked questions relating to sexual function, physical activity, pelvic organ prolapse, maternal satisfaction, ability to feed the baby according to mother's preference, ability to care for baby, social isolation, ability to care for older children (if relevant), impact on seeking healthcare, future pregnancy and mode of birth preference.

Where questionnaires indicate that a woman had to seek medical care related to her CRPT, further data will be collected by reviewing the secondary care medical records.

Independent data validators will assess a proportion of the data from included to assess generalisability, inclusivity and data accuracy via validation of the primary outcome and critical data items.

Safety considerations and assessment of risk

CHAPTER is a cohort study, therefore any interventions that CHAPTER participants undergo will be as part of their routine clinical care. For this reason, there are no safety considerations relating to the

For peer review only - http://bmjopen.bmj.com/site/about/guidelines.xhtml

For peer review only

study. We do not anticipate any risks to study participants. Enrolment in the study will not have any impact on patient care as participants will be receiving the same treatment that they would if they were not enrolled. There is a potential benefit for participants in that contact with a member of the research team may identify a woman experiencing concerning symptoms and/or signs of a physical or mental illness. Any women identified through questionnaires as being particularly distressed will be contacted and offered support by the research team with signposting to appropriate local services. Safeguarding elements related to significant concerns related to physical or mental health will involve contact with local services (primary and/or secondary care as appropriate).

Study participants may find the follow-up contacts for completion of the questionnaires an inconvenience given that the follow-up period continues up to 12 months post study entry. We will mitigate any potential risk of attrition bias by ensuring that participants fully understand the time commitment at enrolment stage and that they know they may withdraw at any time. In accordance with the University of Birmingham standard operating procedures, this study has been risk assessed to clarify any risks relating uniquely to this study beyond that associated with usual care. A risk assessment has been conducted and concluded that this study presents no higher risk than the risk of standard medical care.

Women and clinicians may become distressed when completing the questionnaires. We have therefore developed care pathways within the study to manage this (S2 Appendix) [20]. For participants who have completed the questionnaires electronically we will ask them if they have found the questionnaires distressing and would like additional support from the CHAPTER team. Should participants indicate distress and would like a telephone call, they will be contacted within seven days and a member of the team will direct them towards appropriate postnatal support services and their GP. If a women experiences distress while completing the questionnaires with the clinician over the telephone she will be offered an opportunity to stop the questionnaire, or postpone to a later point and directed to support services as above. Should the clinicians conducting the telephone questionnaires, who are trained midwives/obstetricians experienced in the care of postnatal women with perineal

For peer review only - http://bmjopen.bmj.com/site/about/guidelines.xhtml

trauma, become distressed by women's experiences and/or symptoms, they will be offered support within the CHAPTER team though the chief investigator, in keeping with the normal clinical pathways for doctors and midwives working in the NHS.

Data management

Data entry will be completed by the sites via a bespoke BiCOPS REDCaP study database. The data capture system will conduct automatic range checks for specific data values to ensure high levels of data quality. Queries and requests for missing clinical data will be raised using data clarification forms via the study database, with the expectation that these queries will be completed by the site within 30 days of receipt.

Participant questionnaires will be completed online, by post or telephone according to participant preference. At the time of consent and entry into CHAPTER, what the study involves will be discussed with each woman. It will be explained that completion of quality-of-life questionnaires plus other patient-reported outcomes will be requested until 12 months postpartum and the methods of completion of the questionnaires discussed. Participants who have not returned completed forms will be contacted up to three times after the due date to request completion of the questionnaires. After this time, unreturned forms will be regarded as missing and will not be requested again. At the six week time-point, participant questionnaires can be completed at six weeks postpartum, +/- one week. At the six and 12 month time-points, participant questionnaires can be completed +/- one month.

University of Birmingham has policies in place, which are designed to protect the security, accuracy, integrity and confidentiality of personal data. The study is registered with the Data Protection Officer at the University of Birmingham and will hold data in accordance with the Data Protection Act (2018 and subsequent amendments). The CHAPTER Study Office has arrangements in place for the secure storage and processing of the study data which comply with University of Birmingham policies.

For peer review only - http://bmjopen.bmj.com/site/about/guidelines.xhtml

It is the responsibility of the PI to ensure all essential study documentation and source documents (e.g., signed consent forms, Investigator Site Files, participants' hospital notes) at their site are securely retained for the contractual period. Archiving will be authorised by BiCOPS following submission of the end of study report. No documents should be destroyed without prior approval from the BiCOPS Director. The electronic study master file will be stored at BiCOPS for at least three years after the end of the study. Long-term offsite data archiving facilities will be considered for storage after this time; data will be stored securely and confidentially for at least 25 years.

End of study definition

The end of study will be when the last enrolled participant completes their 12-month questionnaires, all database queries have been resolved and the database has been locked and analyses completed. The CHAPTER Study Office will notify the Research Ethics Council (REC) and the Sponsor within 90 days of the end of study. The CHAPTER Study Office will provide the REC and the Sponsor with a summary report of the study.

Data analyses and statistical considerations

Summary statistics: Simple descriptive statistics will be used to summarize characteristics and outcomes, with categorical variables expressed as percentages and continuous variables as mean averages alongside the corresponding standard deviation. To allow for hospital variation, differences in primary and secondary outcomes by participant characteristics will be assessed using multilevel logistic and linear regression models; unadjusted and adjusted models, using clinically plausible explanatory variables, will be reported. Further analysis will investigate potential sub-groups, identified from our ongoing systematic reviews.

Explanatory variables/characteristics: We plan to explore the risk of our outcomes adjusted for variables deemed potentially significant. These will be identified in the following ways: other work packages within the programme grant related to primary care date and systematic reviews of existing literature, patient and public involvement and clinical co-applicants.

For peer review only - http://bmjopen.bmj.com/site/about/guidelines.xhtml

Status and timeline of study

The CHAPTER cohort study started participant recruitment on 1st September 2023 and has undergone two rounds of recruitment to-date (correct as of 19th March 2024). Questionnaires have followed at the initial six month time-point and are currently being distributed at the six month time-point. The 12 month questionnaires will follow accordingly from 1st September 2024 onwards.

Data availability

Requests for data generated during this study will be considered by BiCOPS. Data will typically be available six months after the primary publication. Only scientifically sound proposals from appropriately qualified Research Groups will be considered for data sharing. The request will be reviewed by the BiCOPS Data Sharing Committee in discussion with the CI and, where appropriate (or in absence of the CI) any of the following: the study sponsor, the study management group, and the programme steering committee. A formal Data Sharing Agreement (DSA) may be required between respective organisations once release of the data is approved and before data can be released. Data will be fully de-identified (anonymised) unless the DSA covers transfer of participant identifiable information.

Patient and public involvement statement

Patient and public involvement has been key to the CHAPTER project. We have a patient advisory group (PAG) who have been providing guidance on the development and initiation of this cohort study. The PAG have been instrumental in developing study materials, ethical considerations and in guiding decisions about rounds of recruitment needed. The PAG will continue to guide all aspects of the CHAPTER project development.

For peer review only - http://bmjopen.bmj.com/site/about/guidelines.xhtml

Ethics and dissemination

Ethical approval has been granted by the Research Ethics Council with reference 23/WA/0169. It is anticipated that minor amendments may need to be submitted after commencement of the study. These will be dealt with via submission to the ethics board and subsequently communicating this amendment to sites to ensure their consent is in-place.

Results of the study will ultimately be submitted for publication in a peer reviewed journal after study completion. Findings will also be submitted to national and international conferences as well as disseminated to the Department of Health and Royal Colleges. Appropriate resources for patients will be developed and any relevant findings are expected to be rapidly incorporated into guidelines by the Royal College of Obstetricians and Gynaecologists, the Royal College of Midwives and NICE.

Word count 3,976

Acknowledgments

This project is part of the CHAPTER program grant and therefore a larger group of collaborators have contributed to the project and are detailed below as part of the CHAPTER group.

**CHAPTER group:

Laura Jones- 1. Institute of Applied Health Research, University of Birmingham, UK

Sara Webb- 1. Royal College of Midwives, UK

John Maltby- 1. School of Psychology and Vision Sciences, University of Leicester, UK

Sarah Hillman- 1. Warwick Medical School, UK

Nicola J Adderley- 1. Institute of Applied Health Research, University of Birmingham, UK;

2. National Institute for Health and Care Research (NIHR) Birmingham Biomedical Research Centre, UK

For peer review only

For peer review only - http://bmjopen.bmj.com/site/about/guidelines.xhtml

Alice Sitch- 1. Institute of Applied Health Research, University of Birmingham, UK. 2. NIHR Birmingham Biomedical Research Centre, University Hospitals Birmingham NHS Foundation Trust and University of Birmingham, UK

Olalekan Lee Aiyegbusi- 1. Centre for Patient Reported Outcomes Research (CPROR), Institute of Applied Health Research, University of Birmingham, UK. 2. NIHR Birmingham Biomedical Research Centre (BRC), University Hospitals Birmingham NHS Foundation Trust and University of Birmingham, UK. 3. NIHR Applied Research Collaboration (ARC) West Midlands, University of Birmingham, Birmingham, UK. 4. NIHR Blood and Transplant Research Unit (BTRU) in Precision Transplant and Cellular Therapeutics, University of Birmingham, Birmingham, UK.

Marian Knight- 1. National Perinatal Epidemiology Unit, University of Oxford, UK

Krishnarajah Nirantharakumar- 1. Institute of Applied Health Research, University of Birmingham, UK 2. NIHR Birmingham Biomedical Research Centre, UK; 3. Health Data Research UK (HDRUK), London, UK

Jane Whitehurst- 1. NIHR Applied Research Collaboration West Midlands, UK

Amie Wilson- 1. Institute of Metabolism and Systems Research, University of Birmingham, UK

Competing interests statement

None known

Funding statement

This project is funded by the National Institute for Health and Care Research (NIHR), grant number NIHR202869. The views expressed are those of the authors and not necessarily those of NIHR or the Department of Health and Social Care.

For peer review only

Author contributions

VHM- initial protocol development, design of methodology, overseeing protocol revisions, RM- writing protocol for submission, editing and revisions, RP- project administration, TH- data curation, project administration, ST- project administration, CM- conceptualization, design of methodology, drafting protocol, LM- conceptualization, design of methodology, RKM- initial protocol development, conceptualization, funding acquisition, design of methodology. CHAPTER group: conceptualization, funding acquisition, methodology. All authors read and approved the final manuscript.

For peer review only

References

1. NHS Digital. NHS Maternity Statistics 2018-2019 [online]. 2019. https://files.digital.nhs.uk/D0/C26F84/hosp-epis-stat-mat-summary-report-2018-19.pdf (accessed 10 August 2023).

2. Smith LA, Price N, Simonite V, et al. Incidence of and risk factors for perineal trauma: a prospective observational study. BMC Pregnancy Childbirth. 2013;13(1): 59. doi: 10.1186/1471-2393-13-59

3. Andrews V, Thakar R, Sultan AH, et al. Evaluation of postpartum perineal pain and dyspareunia—a prospective study. Eur J Obstet Gynecol Reprod Biol. 2008;137(2):152-156.

4. Leeman L, Rogers R, Borders N, et al. The effect of perineal lacerations on pelvic floor function and anatomy at 6 months postpartum in a prospective cohort of nulliparous women. Birth. 2016;43(4): 293-302.

5. Dudley L, Kettle C, Ismail K. Prevalence, pathophysiology and current management of dehisced perineal wounds following childbirth. Br J Midwifery. 2013;21(3):160-171.

6. McDonald E, Gartland D, Small R, et al. Dyspareunia and childbirth: a prospective cohort study. BJOG. 2015;122(5): 672-679.

7. Opondo C, Harrison S, Sanders J, et al. The relationship between perineal trauma and postpartum psychological outcomes: a secondary analysis of a population-based survey. BMC Pregnancy Childbirth. 2023;23(1): 639. doi: 10.1186/s12884-023-05950-6.

For peer review only - http://bmjopen.bmj.com/site/about/guidelines.xhtml

8. Priddis H, Schmied V, Dahlen H. Women's experiences following severe perineal trauma: a qualitative study. BMC Womens Health. 2014;14(1): 32. doi: 10.1186/1472-6874-14-32.

9. National Institute for Health and Clinical Excellence. Postnatal Care [online]. 2021. https://www.nice.org.uk/guidance/ng194 (accessed 11 August 2023).

10. NMPA Project Team. National maternity and perinatal audit: clinical report 2019 [online]. 2019. https://www.hqip.org.uk/resource/national-maternity-and-perinatal-audit-nmpa-clinical-report-2019/ (accessed 1 March 2024).

11. Knight M, Chiocchia V, Partlett C, et al. Prophylactic antibiotics in the prevention of infection after operative vaginal delivery (ANODE): a multicentre randomised controlled trial. Lancet. 2019;393(10189): 2395-2403.

12. Hodgetts-Morton V, Hewitt CA, Wilson A, et al. Vaginal preparation with chlorhexidine at cesarean section to reduce endometritis and prevent sepsis: A randomized pilot trial (PREPS). Acta Obstet Gynecol Scand. 2020;99(2): 231-239.

13. Sansoni JE, Marosszeky N, Sansoni E, et al. The development of the revised urinary incontinence scale (RUIS): Australian health outcomes collaboration [online]. 2008. https://ro.uow.edu.au/gsbpapers/64 (accessed 22 February 2024).

14. Sansoni J, Hawthorne G, Fleming G, et al. The revised faecal incontinence scale: a clinical validation of a new, short measure for assessment and outcomes evaluation. Dis Colon Rectum. 2013;56(5): 652-659.

15. Cox JL, Holden JM, Sagovsky R. Detection of postnatal depression: development of the 10-item edinburgh postnatal depression scale. Br J Psychiatry. 1987;150(6): 782-786.

16. Herdman M, Gudex C, Lloyd A, et al. Development and preliminary testing of the new five-level version of EQ-5D (EQ-5D-5L). Qual Life Res. 2011;20(10): 1727-1736.

17. Ayers S, Wright DB, Thornton A. Development of a measure of postpartum PTSD: the city birth trauma scale. Front Psychiatry. 2018;9. doi: 10.3389/fpsyt.2018.00409.

18. Barber MD, Kuchibhatla MN, Pieper CF, et al. Psychometric evaluation of 2 comprehensive condition-specific quality of life instruments for women with pelvic floor disorders. Am J Obstet Gynecol. 2001;185(6): 1388-1395.

For peer review only - http://bmjopen.bmj.com/site/about/guidelines.xhtml

19. Thibault-Gagnon S, Yusuf S, Langer S, et al. Do women notice the impact of childbirth-related levator trauma on pelvic floor and sexual function? Results of an observational ultrasound study. Int Urogynecol J. 2014;25(10): 1389-1398.

20. Draucker CB, Martsolf DS, Poole C. Developing distress protocols for research on sensitive topics. Arch Psychiatr Nurs. 2009;23(5): 343-350.

Supporting information captions :

S1 Appendix- 6 week questionnaire

S2 Appendix- distress pathway

For peer review only

For peer review only - http://bmjopen.bmj.com/site/about/guidelines.xhtml

S1 Appendix: Six week Quality of life questionnaire

Date added to REDCap 6 week QoL __________________________________

6 Week QoL Questionnaire

You kindly agreed to take part in the Chapter cohort study following the birth of your baby. This research aims to understand how common complications are amongst women who have experienced childbirth acquired perineal trauma. To do this, we would like to collect some information from you at 6 weeks, 6 months and 12 months following the birth of your baby.

We have used three words frequently in the questionnaire - perineum, stitches and tear. Perineum is the part of your body between the opening of your vagina and your back passage. Stitches and tear refer to the cuts or tears in your perineum or vaginal area (either made by the midwife/doctor or happening on their own) during the birth of your baby.The perineum connects with the muscles in the lower pelvis to form the pelvic floor. Your pelvic floor helps to control your bladder, bowel and sexual function.

We expect the questionnaire will take you approximately 20 minutes to complete. It is divided into 4 sections:

Section 1: This section asks in detail about the healing of your stitches/tear and how satisfied you felt about the support you received from healthcare professionals in relation to the healing of your perineum. It asks whether you have had an infection or any complications and whether you have needed to be readmitted to hospital for treatment.

Section 2: This section explores if any symptoms related to the healing of your stitches/tear may have impacted the experience of feeding and caring for your baby (and older children, if applicable).

Section 3: This section will focus on specific questions relating to whether there have been any changes to your pelvic floor and how this impacts your bladder and bowel function. It also asks how these changes influence your day-to-day life.

Section 4: The last part of the questionnaire asks several questions about your mental health and wellbeing.

Within the Chapter study we use the term woman to refer to all birthing people as we acknowledge that it is not only people who identify as women who may be affected by childbirth related perineal trauma.

Sharing your experiences with us will increase our knowledge and lead to better understanding in this area. All the information that you provide will be confidential and may help us to improve the care women receive following childbirth.

By completing this questionnaire, you are confirming that you are still happy to take part in this research. If you decide you wish to withdraw from this study or have any queries, please contact the Chapter study research team on chapter@contacts.bham.ac.uk.

If at any time you feel this questionnaire is distressing you. you can use the 'save & return later' button to leave. If you wish you can return and complete the rest of the questionnaire at a more convenient time.

Thank you again for your participation and please remember that we are more than happy for you to contact us if you need any assistance with completing the questionnaire.

For peer review only - http://bmjopen.bmj.com/site/about/guidelines.xhtml

Section 1: This section asks in detail about the healing of your stitches/tear and how satisfied you felt about the support you received from healthcare professionals in relation to the healing of your perineum. It asks whether you have had an infection or any complications and whether you have needed to be readmitted to hospital for treatment.

Have you had any problems with the healing of your stitches or tear?

◯ Yes ◯ No

Since the birth of your baby have you noticed any discharge or leakage of fluid from any part of the stitches/tear?

◯ Yes ◯ No

Was the discharge or leakage either:

◯ Clear or blood stained
◯ Yellow/ green (pus)
◯ Other,please specify

Describe discharge or leakage:

____

Please tick any of the following additional symptoms that apply to your stitches/ tear: ______

______

When did you first notice any of these symptoms?

◯ 0-2 weeks after having my baby
◯ 2-4 weeks after having my baby
◯ 4-6 weeks after having my baby
◯ Unsure

Please check you have not missed any questions above before you continue to the next section. If you do not want to answer any questions for any personal reasons, that is fine.

Did you see a health care professional for any problems connected with your stitches or tear during this time?

◯ Yes ◯ No

Please indicate which health care professional you saw in the period following the birth of your baby for any problems connected with your stitches/tear

Doctor in hospital

◯ Yes ◯ No

How satisfied you were with the support you were given in relation to the healing of your stitches/tear from the Doctor at hospital

◯ Very satisfied
◯ Satisfied
◯ Unsatisfied
◯ Very unsatisfied

For peer review only

How satisfied you were with the support you were given in relation to the healing of your stitches/tear from the Doctor at hospital

◯ Very satisfied
◯ Satisfied
◯ Neutral
◯ Unsatisfied
◯ Very unsatisfied

Midwife at hospital

◯ Yes ◯ No

How satisfied you were with the support you were given in relation to the healing of your stitches/tear from the Midwife

◯ Very satisfied
◯ Satisfied
◯ Unsatisfied
◯ Very unsatisfied

How satisfied you were with the support you were given in relation to the healing of your stitches/tear from the Midwife

◯ Very satisfied
◯ Satisfied
◯ Neutral
◯ Unsatisfied
◯ Very unsatisfied

Midwife in community

◯ Yes ◯ No

How satisfied you were with the support you were given in relation to the healing of your stitches/tear from the Midwife

◯ Very satisfied
◯ Satisfied
◯ Unsatisfied
◯ Very unsatisfied

How satisfied you were with the support you were given in relation to the healing of your stitches/tear from the Midwife

◯ Very satisfied
◯ Satisfied
◯ Neutral
◯ Unsatisfied
◯ Very unsatisfied

Your General Practioner (GP)

◯ Yes ◯ No

For peer review only

How satisfied you were with the support you were given in relation to the healing of your stitches/tear from the GP

◯ Very satisfied
◯ Satisfied
◯ Unsatisfied
◯ Very unsatisfied

How satisfied you were with the support you were given in relation to the healing of your stitches/tear from the GP

◯ Very satisfied
◯ Satisfied
◯ Neutral
◯ Unsatisfied
◯ Very unsatisfied

Health visitor

◯ Yes ◯ No

How satisfied you were with the support you were given in relation to the healing of your stitches/tear from the Health visitor

◯ Very satisfied
◯ Satisfied
◯ Unsatisfied
◯ Very unsatisfied

How satisfied you were with the support you were given in relation to the healing of your stitches/tear from the Health visitor

◯ Very satisfied
◯ Satisfied
◯ Neutral
◯ Unsatisfied
◯ Very unsatisfied

Physiotherapist at the hospital

◯ Yes ◯ No

How ssatisfied you were with the support you were given in relation to the healing of your stitches/tear from the Physiotherapist

◯ Very satisfied
◯ Satisfied
◯ Unsatisfied
◯ Very unsatisfied

How ssatisfied you were with the support you were given in relation to the healing of your stitches/tear from the Physiotherapist

◯ Very satisfied
◯ Satisfied
◯ Neutral
◯ Unsatisfied
◯ Very unsatisfied

For peer review only

Private physiotherapy

◯ Yes ◯ No

How satisfied you were with the support you were given in relation to the healing of your stitches/tear from the private Physiotherapist

◯ Very satisfied
◯ Satisfied
◯ Unsatisfied
◯ Very unsatisfied

How satisfied you were with the support you were given in relation to the healing of your stitches/tear from the private Physiotherapist

◯ Very satisfied
◯ Satisfied
◯ Neutral
◯ Unsatisfied
◯ Very unsatisfied

Other heathcare professional

◯ Yes ◯ No

Confirm which other healthcare professional you have received advice and support from:

____

How satisfied you were with the support you were given in relation to the healing of your stitches/tear from the [anode_hcw_other_name]

◯ Very satisfied
◯ Satisfied
◯ Unsatisfied
◯ Very unsatisfied

How satisfied you were with the support you were given in relation to the healing of your stitches/tear from the [anode_hcw_other_name]

◯ Very satisfied
◯ Satisfied
◯ Neutral
◯ Unsatisfied
◯ Very unsatisfied

You have indicated that you didn't seek any further assistance from a health care professional in relation to your stitches/tear. Please indicate why you did not seek assistance. You can tick more than one - please tick ALL that apply.

☐ I didn't feel I needed to as I recovered well after the birth
☐ I wasn't aware of any services that could help me
☐ I was too embarrassed to discuss my symptoms and concerns
☐ I was fearful about an intimate examination
☐ I had some concerns and knew services existed but I haven't been able to find the time to seek further assistance
☐ Other

PLease specify Other reason you did not seek assistance.

____

For peer review only

Please check you have not missed any questions above before you continue to the next section. If you do not want to answer any questions for any personal reasons, that is fine.

Hospital Visits

Did you have to go to a hospital clinic as an out-patient for any problems connected with your stitches/tear since the birth of your baby?

◯ Yes ◯ No

What was the reason you attended the hospital as an out-patient? You can tick more than one - please tick ALL that apply.

☐ Pain or soreness in addition to the discomfort experienced following the birth
☐ Redness or inflammation spreading from the edges of the stitches/tear
☐ The area around the stitches/tear felt warmer/hotter than the surrounding skin
☐ The area around the stitches/tear became swollen
☐ The edges of any part of the stitches/tear separated or gaped open
☐ Other, please specify

Confirm the other reason you had to go to out-patients

____

How many times have you had to go to out-patients

____

Please check you have not missed any questions above before you continue to the next section. If you do not want to answer any questions for any personal reasons, that is fine.

Have you been readmitted to hospital for any reason connected with your own health since the birth of your baby? (We don't need to know if you were admitted with your baby because your baby was unwell)

◯ Yes ◯ No

How many times have you been readmitted for any reason connected with your own health since the birth of your baby?

◯ 1 ◯ 2 ◯ 3 ◯ more than 3

Please tell us about your first readmission to hospital by providing the following details.

Name of hospital of 1st re-admission

____

For peer review only

What was the reason for this first readmission? You can tick more than one - please tick ALL that apply.

☐ Pain or soreness in addition to the discomfort experienced following the birth
☐ Redness or inflammation spreading from the edges of the stitches/tear
☐ The area around the stitches/tear felt warmer/hotter than the surrounding skin
☐ The area around the stitches/tear became swollen
☐ The edges of any part of the stitches/tear separated or gaped open
☐ Other, please specify

Please specify Other reason for 1st re-admission

____

How long after you gave birth to your baby were you readmitted to hospital the first time?

◯ Less than 1 week
◯ 1-2 weeks
◯ 2-3 weeks
◯ 3-4 weeks
◯ 4-5 weeks
◯ 5-6 weeks

Did you have an operation when you were readmitted the first time?

◯ Yes ◯ No

Were you in the intensive care unit when you were readmitted the first time?

◯ Yes ◯ No

How many nights did you stay in hospital during this first readmission?

____

Please tell us about your second readmission to hospital by providing the following details.

Name of hospital of 2nd re-admission

____

What was the reason for this second readmission? You can tick more than one - please tick ALL that apply.

☐ Pain or soreness in addition to the discomfort experienced following the birth
☐ Redness or inflammation spreading from the edges of the stitches/tear
☐ The area around the stitches/tear felt warmer/hotter than the surrounding skin
☐ The area around the stitches/tear became swollen
☐ The edges of any part of the stitches/tear separated or gaped open
☐ Other, please specify

Please specify Other reason for 2nd re-admission

____

For peer review only - http://bmjopen.bmj.com/site/about/guidelines.xhtml

How long after you gave birth to your baby were you readmitted to hospital the second time?

◯ Less than 1 week
◯ 1-2 weeks
◯ 2-3 weeks
◯ 3-4 weeks
◯ 4-5 weeks
◯ 5-6 weeks

Did you have an operation when you were readmitted the second time?

◯ Yes ◯ No

Were you in the intensive care unit when you were readmitted the first time?

◯ Yes ◯ No

How many nights did you stay in hospital during this second readmission?

____

Please tell us about your third readmission to hospital by providing the following details.

Name of hospital of 3rd re-admission

____

What was the reason for this third readmission? You can tick more than one - please tick ALL that apply.

☐ Pain or soreness in addition to the discomfort experienced following the birth
☐ Redness or inflammation spreading from the edges of the stitches/tear
☐ The area around the stitches/tear felt warmer/hotter than the surrounding skin
☐ The area around the stitches/tear became swollen
☐ The edges of any part of the stitches/tear separated or gaped open
☐ Other, please specify

Please specify Other reason for 3rd re-admission

____

How long after you gave birth to your baby were you readmitted to hospital the third time?

◯ Less than 1 week
◯ 1-2 weeks
◯ 2-3 weeks
◯ 3-4 weeks
◯ 4-5 weeks
◯ 5-6 weeks

Did you have an operation when you were readmitted the third time?

◯ Yes ◯ No

For peer review only

Were you in the intensive care unit when you were readmitted the third time?

◯ Yes ◯ No

How many nights did you stay in hospital during this third readmission?

____

As you have been re-admitted to hospital more than 3 times please write how many ADDITIONAL nights you have stayed

____

Please check you have not missed any questions above before you continue to the next section. If you do not want to answer any questions for any personal reasons, that is fine.

Have you been prescribed any antibiotics since the birth of your baby? (if you were readmitted to hospital include any antibiotics you were given then).

◯ Yes
◯ No
◯ Unknown drug received
◯ Cannot remember if any medication was received

What were they given for?

☐ Pain or soreness in addition to the discomfort experienced following the birth
☐ Redness or inflammation spreading from the edges of the stitches/tear
☐ The area around the stitches/tear felt warmer/hotter than the surrounding skin
☐ The area around the stitches/tear became swollen
☐ The edges of any part of the stitches/tear separated or gaped open
☐ Other, please specify

Other reason drugs were given

____

Who prescribed them?- please tick ALL that apply.

☐ GP
☐ Doctor in maternity unit
☐ Doctor in emergency department
☐ Other

Other prescriber, please specify

____

Were they prescribed more than 24 hours after the birth of your baby?

◯ Yes ◯ No

Please check you have not missed any questions above before you continue to the next section. If you do not want to answer any questions for any personal reasons, that is fine.

For peer review only

Has your doctor taken any bloods or swabs to send to the lab to check for infection

◯ Yes
◯ No
◯ Don't know

Have you been told you had an infection in your perineum

◯ Yes
◯ No
◯ Unsure

Please tick all signs and symptoms you experienced:

☐ Fever >38 degrees
☐ Perineal pain
☐ Smelly discharge
☐ Redness around the stiches/tear
☐ Swelling
☐ Other

Other signs and symptoms you experienced:

____

Please check you have not missed any questions above before you continue to the next section. If you do not want to answer any questions for any personal reasons, that is fine.

In the past week, have you experienced any pain or unpleasant feeling in your perineum? (the area between your vagina and back passage)

◯ Yes ◯ No

Would you describe the strength of the pain or unpleasant feeling as?

◯ Mild
◯ Moderate
◯ Severe

Is the pain or unpleasant feeling there?

◯ Some of the time
◯ Most of the time
◯ All of the time

In the past week have you needed any tablets to relieve the pain or discomfort in your perineum?

◯ Yes ◯ No

Please check you have not missed any questions above before you continue to the next section. If you do not want to answer any questions for any personal reasons, that is fine.

For peer review only

How well do you feel that your perineum has healed? . (Please tick one box that is the most appropriate statement for the way you feel)

◯ I feel that my perineum has healed well
◯ I feel that my perineum has healed satisfactorily
◯ I feel that my perineum has healed poorly
◯ I feel that my perineum has healed very poorly

Please check you have not missed any questions above before you continue to the next section. If you do not want to answer any questions for any personal reasons, that is fine.

For peer review only

Section 2: This section explores if any symptoms related to the healing of your stitches/tear may have impacted the experience of feeding and caring for your baby (and older children, if applicable).

Have you breastfed your baby at any time since he/she was born

◯ Yes ◯ No

Are you still breastfeeding your baby

◯ Yes ◯ No

If you are no longer breastfeeding your baby: how old was your baby when you stopped breastfeeding? Please specify to the closest week breastfeeding stopped.

◯ Less than 1 week
◯ Between 1-2 weeks
◯ Between 2-3 weeks
◯ Between 3-4 weeks
◯ Between 4-5 weeks
◯ Between 5-6 weeks

Why did you stop breastfeeding You can tick more than one - please tick ALL that apply.

☐ Painful nipples
☐ Engorgement
☐ Blocked milk duct
☐ Thrush
☐ Mastitis
☐ Breast abscess
☐ Not enough milk
☐ Perineum was too uncomfortable or painful whilst feeding my baby
☐ Other

Other reason stopped

____

Has your perineum been too uncomfortable or painful for you to feed your baby

◯ Yes ◯ No

Have problems or worries about your perineum caused you to not be able to care for your baby in the way you'd like to during this time? (Care includes washing/ feeding/ playing/ holding your baby).

◯ Yes
◯ No
◯ I have not had any problems or worries about my perineum

How often do these problems or worries about your perineum interfere with you caring for your baby:

◯ A lot of the time (for example, more than once a day)
◯ Some of the time (for example, at least once a day)
◯ Not very often

For peer review only

Do you feel problems or worries about your perineum have affected bonding with your baby during this time?

◯ Yes
◯ No
◯ I have not had any problems or worries about my perineum

Have problems or worries about your perineum caused you to not be able to care for your older children in the way you'd like?

◯ Yes
◯ No
◯ I don't have any older children

How often do these problems or worries about your perineum interfere with you caring for your older child?

◯ A lot of the time (for example, more than once a day)
◯ Some of the time (for example, at least once a day)
◯ Not very often

Please check you have not missed any questions above before you continue to the next section. If you do not want to answer any questions for any personal reasons, that is fine.

For peer review only

Section 3: This section will focus on specific questions relating to whether there have been any changes to your pelvic floor and how this impacts your bladder and bowel function. It also asks how these changes influence your relationships and day-to-day life

Please consider your experiences during the last 4 weeks for the following.

Have you had any problems with controlling your bowels? (This includes passing of wind (flatus) and stool).

◯ Yes ◯ No

Do you have difficulty controlling wind (flatus)?

◯ Never
◯ Rarely (for example less than once in the past 4 weeks)
◯ Sometimes (for example less than once a week, but once or more in the past 4 weeks)
◯ Often or usually (for example less than once a day but once a week or more)
◯ Always (for example once or more per day or whenever you have a bowel movement)

Do you leak, have accidents or lose control with solid stool

◯ Never
◯ Rarely i.e. less than once in the past four weeks
◯ Sometimes i.e. less than once a week, but once or more in the past four weeks
◯ Often or usually i.e. less than once a day but once a week or more
◯ Always i.e. once or more per day or whenever you have a bowel movement

Do you leak, have accidents or lose control with liquid stool

◯ Never
◯ Rarely i.e. less than once in the past four weeks
◯ Sometimes i.e. less than once a week, but once or more in the past four weeks
◯ Often or usually i.e. less than once a day but once a week or more
◯ Always i.e. once or more per day or whenever you have a bowel movement

Do you leak stool if you don't get to the toilet in time?

◯ Never
◯ Rarely i.e. less than once in the past four weeks
◯ Sometimes i.e. less than once a week, but once or more in the past four weeks
◯ Often or usually i.e. less than once a day but once a week or more
◯ Always i.e. once or more per day or whenever you have a bowel movement

Does stool leak so that you have to change your underwear

◯ Never
◯ Rarely i.e. less than once in the past four weeks
◯ Sometimes i.e. less than once a week, but once or more in the past four weeks
◯ Often or usually i.e. less than once a day but once a week or more
◯ Always i.e. once or more per day or whenever you have a bowel movement

Does bowel or stool leakage cause you to alter your lifestyle

◯ Never
◯ Rarely i.e. less than once in the past four weeks
◯ Sometimes i.e. less than once a week, but once or more in the past four weeks
◯ Often or usually i.e. less than once a day but once a week or more
◯ Always i.e. once or more per day or whenever you have a bowel movement

Please check you have not missed any questions above before you continue to the next section. If you do not want to answer any questions for any personal reasons, that is fine.

For peer review only

Please consider your experiences during the last 4 weeks for the following.

Have you had any problems with controlling your bladder?

◯ Yes ◯ No

Do you experience, and if so, how much are you bothered by:

Urine leakage related to the feeling of urgency

◯ Not at all
◯ Slightly
◯ Moderately
◯ Greatly

Urine leakage related to physical activity, coughing or sneezing

◯ Not at all
◯ Slightly
◯ Moderately
◯ Greatly

Small amounts of urine leakage (drops)

◯ Not at all
◯ Slightly
◯ Moderately
◯ Greatly

How often do you experience urine leakage

◯ Never
◯ Less than once a month
◯ A few times a month
◯ A few times a week
◯ Every day and/or night

How much urine do you lose each time

◯ None
◯ Drops
◯ Small splashes
◯ More

Please check you have not missed any questions above before you continue to the next section. If you do not want to answer any questions for any personal reasons, that is fine.

Under each heading, please select the ONE box that best describes your health TODAY

MOBILITY

◯ I have no problems in walking about
◯ I have slight problems in walking about
◯ I have moderate problems in walking about
◯ I have severe problems in walking about
◯ I am unable to walk about

For peer review only

SELF-CARE

◯ have no problems washing or dressing myself
◯ I have slight problems washing or dressing myself
◯ I have moderate problems washing or dressing myself
◯ I have severe problems washing or dressing myself
◯ I am unable to wash or dress myself

USUAL ACTIVITIES (e.g. work, study, housework, family or leisure activities)

◯ I have no problems doing my usual activities
◯ I have slight problems doing my usual activities
◯ I have moderate problems doing my usual activities
◯ I have severe problems doing my usual activities
◯ I am unable to do my usual activities

PAIN / DISCOMFORT

◯ I have no pain or discomfort
◯ I have slight pain or discomfort
◯ I have moderate pain or discomfort
◯ I have severe pain or discomfort
◯ I have extreme pain or discomfor

ANXIETY / DEPRESSION

◯ I am not anxious or depressed
◯ I am slightly anxious or depressed
◯ I am moderately anxious or depressed
◯ I am severely anxious or depressed
◯ I am extremely anxious or depressed

We would like to know how good or bad your health is TODAY. This scale is numbered from 0 to 100.
100 means the best health you can imagine.
0 means the worst health you can imagine.
Please click on the scale to indicate how your health is TODAY.

0 - The worst health you can imagine — 50 — 100 - The best health you can imagine

(Place a mark on the scale above)

Please check you have not missed any questions above before you continue to the next section. If you do not want to answer any questions for any personal reasons, that is fine.

For peer review only

Section 4: The last part of the questionnaire asks several questions about your mental health and wellbeing.

While it is common to feel a little bit low after having a baby, we would like you to know that there are people who can provide support or information if you are struggling to cope with this.

Getting the right help from a health professional early on leads to a faster recovery. Please speak to your GP or health visitor if there is anything that is troubling you and they will be able to support you. You may also find some of the resources at the end of this questionnaire helpful too.

In the below questions please select the answer that comes closest to how you have felt IN THE PAST 7 DAYS, not just how you feel today.

I have been able to laugh and see the funny side of things

◯ As much as I always could
◯ Not quite so much now
◯ Definitely not so much now
◯ Not at all

I have looked forward with enjoyment to things

◯ As much as I ever did
◯ Rather less than I used to
◯ Definitely less than I used to
◯ Hardly at all

I have blamed myself unnecessarily when things went wrong

◯ Yes, most of the time
◯ Yes, some of the time
◯ Not very often
◯ No, never

I have been anxious or worried for no good reason

◯ No, not at all
◯ Hardly ever
◯ Yes, sometimes
◯ Yes, very often

I have felt scared or panicky for no very good reason

◯ Yes, quite a lot
◯ Yes, sometimes
◯ No, not much
◯ No, not at all

Things have been getting on top of me

◯ Yes, most of the time I haven't been able to cope at all
◯ Yes, sometimes I haven't been coping as well as usual
◯ No, most of the time I have coped quite well
◯ No, I have been coping as well as ever

Please check you have not missed any questions above before you continue to the next section. If you do not want to answer any questions for any personal reasons, that is fine.

For peer review only

This following questions asks about your experience during the birth of your most recent baby. It asks about potential traumatic events during (or immediately after) the labour and birth, and whether you are experiencing symptoms that are reported by some women after birth and how often you have experienced these in the last week. Please tick the responses closest to your experience.

Recurrent unwanted memories of the birth (or parts of the birth) that you can't control

- ◯ Not at all
- ◯ Once
- ◯ 2-4 times
- ◯ 5 or more times

Bad dreams or nightmares about the birth (or related to the birth)

- ◯ Not at all
- ◯ Once
- ◯ 2-4 times
- ◯ 5 or more times

Flashbacks to the birth and/or reliving the experience

- ◯ Not at all
- ◯ Once
- ◯ 2-4 times
- ◯ 5 or more times

Getting upset when reminded of the birth

- ◯ Not at all
- ◯ Once
- ◯ 2-4 times
- ◯ 5 or more times

Feeling tense or anxious when reminded of the birth

- ◯ Not at all
- ◯ Once
- ◯ 2-4 times
- ◯ 5 or more times

Trying to avoid things that remind me of the birth (for example, people, places, TV programmes)

- ◯ Not at all
- ◯ Once
- ◯ 2-4 times
- ◯ 5 or more times

Not able to remember details of the birth

- ◯ Not at all
- ◯ Once
- ◯ 2-4 times
- ◯ 5 or more times

For peer review only

Blaming myself or others for what happened during the birth

◯ Not at all
◯ Once
◯ 2-4 times
◯ 5 or more times

Feeling strong negative emotions about the birth (for example, fear, anger, shame)

◯ Not at all
◯ Once
◯ 2-4 times
◯ 5 or more times

When did these symptoms start?

◯ Before the birth
◯ In the first 6 weeks after the birth

Do these symptoms cause you a lot of distress?

◯ Yes
◯ No
◯ Sometimes

How long have these symptoms lasted?

◯ Less than 1 month
◯ 1 to 2 months

Do they prevent you doing things you usually do (for example, socialising, daily activities?)

◯ Yes
◯ No
◯ Sometimes

Could any of these symptoms be due to medication, alcohol, drugs or physical illness?

◯ Yes
◯ No
◯ Sometimes

Please check you have not missed any questions above before you continue to the next section. If you do not want to answer any questions for any personal reasons, that is fine.

You may have found answering some of these questions distressing. The research team can contact you by telephone to offer support or you can contact your GP. Please indicate below whether you would like the research team to contact you.

◯ I would like to receive a telephone call to offer support.
◯ I do not wish to receive a support call.

Additonal Information

Is there anything else you would like to tell us about the healing of your stitches/tear or how you are feeling now? Please feel free to make any comments important to you in this space.

For peer review only

Thank you very much for taking the time to complete this questionnaire. We will be contacting you to ask you to complete the next questionnaire approximately 6 months following the birth of your baby. If you would like to speak to the Chapter research team about any aspect of this study, please contact the team by email chapter@contacts.bham.ac.uk.

Should you need further support or information at any point please speak to your GP or health visitor. You may find the following resources helpful: www.nhs.uk - to find information and advice on health conditions, symptoms and how to get help www.masic.org.uk - a charity which supports women who have sustained severe perineal trauma during childbirth www.mind.org.uk - a charity that provides advice and support to empower anyone experiencing a mental health problem www.pandasfoundation.org.uk - a charity which provides support for every parent who is struggling with their perinatal mental health

For peer review only

S2 Appendix: Distress Pathway

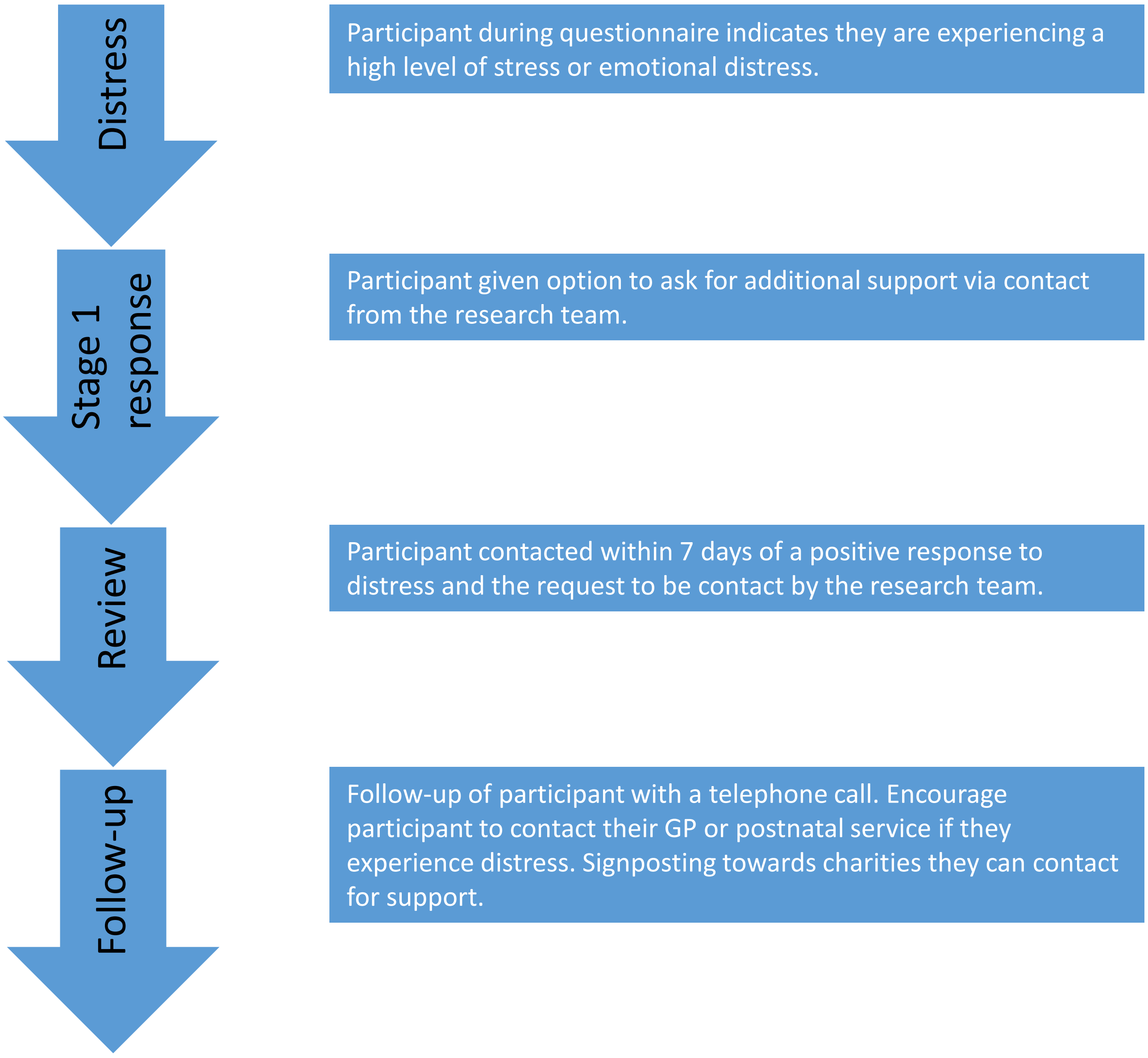

Protocol for managing distress in the context of research on sensitive subjects (adapted from Draucker et al.)

BMJ Open

CHILDBIRTH ACQUIRED PERINEAL TRAUMA STUDY (CHAPTER): A UK PROSPECTIVE COHORT STUDY PROTOCOL

Journal:	*BMJ Open*
Manuscript ID	bmjopen-2024-086724.R1
Article Type:	Protocol
Date Submitted by the Author:	12-Apr-2024
Complete List of Authors:	Hodgetts Morton, Victoria ; Birmingham Women's Hospital; University of Birmingham Institute of Applied Health Research<br>Man, Rebecca; University of Birmingham Institute of Applied Health Research<br>Perry, Rita; University of Birmingham Institute of Applied Health Research<br>Hughes, Terry; University of Birmingham Institute of Applied Health Research<br>Tohill, Susan; University of Birmingham Institute of Applied Health Research<br>MacArthur, Christine; University of Birmingham Institute of Applied Health Research, Public Health<br>Magill, Laura ; University of Birmingham Institute of Applied Health Research<br>Morris, R. K.; Birmingham Women's Hospital; University of Birmingham Institute of Applied Health Research<br>Group, CHAPTER ; The CHAPTER Collaborative
**Primary Subject Heading**:	Obstetrics and gynaecology
Secondary Subject Heading:	Obstetrics and gynaecology
Keywords:	Postpartum Period, Natural Childbirth, Postpartum Women < Postpartum Period, OBSTETRICS, Pregnancy, WOUND MANAGEMENT

SCHOLARONE™
Manuscripts

CHILDBIRTH ACQUIRED PERINEAL TRAUMA STUDY (CHAPTER): A UK PROSPECTIVE COHORT STUDY PROTOCOL

Victoria Hodgetts Morton[1,2], Rebecca Man[1]*, Rita Perry[1], Terry Hughes[1], Susan Tohill[1], Christine MacArthur[1], Laura Magill[1], R Katie Morris[1,2] on behalf of the CHAPTER group**

1. Institute of Applied Health Research, University of Birmingham, Birmingham, United Kingdom

2. Birmingham Women's and Children's NHS Foundation Trust, Birmingham, United Kingdom

*Corresponding author

Email: r.man@bham.ac.uk

Keywords: postpartum period, natural childbirth, pregnant women, postpartum women

For peer review only

For peer review only - http://bmjopen.bmj.com/site/about/guidelines.xhtml

CHILDBIRTH ACQUIRED PERINEAL TRAUMA STUDY (CHAPTER): A UK PROSPECTIVE COHORT STUDY PROTOCOL

Abstract

Introduction

Childbirth related perineal trauma (CRPT) is the commonest complication of childbirth affecting 80% of women overall after vaginal birth. There remains a lack of comprehensive evidence relating to the prevalence of subsequent health problems. Current evidence is related to short-term outcomes, for example pain, but there is less known about longer term outcomes such as infection, wound dehiscence, pelvic floor function and psychological outcomes. This is a protocol for a cohort study assessing outcomes of women after CRPT.

Methods and analysis

A multi-centre, prospective UK cohort study aiming to include 1000 women. All women who have sustained CRPT will be eligible for inclusion and will be followed-up for 12 months after childbirth. The primary outcome will be perineal infection at six weeks post-birth. Secondary outcomes will include antibiotic use for perineal infection, wound breakdown, use of analgesia, requirement for admission or surgical intervention, urinary and faecal incontinence, anxiety and depressive symptoms, sexual function and impact on daily activities. Outcomes will be measured at six weeks, six months and 12 months postpartum, with some outcomes being measured at all time-points and others at selected most appropriate time-points only. Outcome data will be obtained from review of clinical notes and from patient questionnaires. Simple descriptive statistics will be used to summarize characteristics and outcomes, with categorical variables expressed as percentages and continuous variables as mean averages, alongside the corresponding standard deviation.

For peer review only - http://bmjopen.bmj.com/site/about/guidelines.xhtml

Ethics and dissemination

Ethical approval has been granted by the Research Ethics Council with reference 23/WA/0169. Data collected from the Childbirth Acquired Perineal Trauma (CHAPTER) cohort study will highlight the prevalence and type of complications after CRPT and which women are more at risk. After the conclusion of this study, findings will be used to work with governmental organisations and Royal Colleges to target resources and ultimately improve care.

Article Summary

Strengths and limitations

- This study aims to incorporate a diverse range of women with childbirth related perineal trauma (CRPT) from across the UK, with the goal of producing representative and up-to-date information on complications after CRPT.
- Follow-up questionnaires are comprehensive and holistic with a range of potential physical and psychological complications after CRPT being assessed.
- Follow-up for the year after childbirth is integral to this study and we will need to consider the potential for non-response to the questionnaires, particularly at the later data collection points.
- Questionnaires will need to be completed and returned within the specified time-frame in relation to the follow-up points. Where responses occur outside these time windows we will need to consider the impact of this on response.

Introduction

In the UK every year, 80% of women who have a vaginal birth (approximately 450,000 women), experience childbirth related perineal trauma (CRPT) [1, 2]. CRPT affects 91% of women in a first birth and 70% in successive births and is therefore the most common complication after childbirth [2]. Despite CRPT affecting the vast majority of women who give birth, the prevalence and severity of resulting health complications remains unclear. There is some evidence relating to initial outcomes, for example pain,

For peer review only

For peer review only - http://bmjopen.bmj.com/site/about/guidelines.xhtml

but less is known about longer-term complications, including; wound dehiscence, infection, psychological or social issues, sexual dysfunction and incontinence [3-6].

Complications from CRPT can be multi-faceted and long-lasting, with some women suffering wound related complications for many years and ultimately requiring complex reconstructive surgery, or developing long-term psychological problems [7, 8]. There is currently very little research exploring the prevalence of severe CRPT, its risk factors, management or its short or long-term complications [9]. Information to tell us how serious these complications are or how often they occur, is not routinely collected. There is very little research to indicate which women might be more likely to suffer complications from CRPT or experience longer-term health problems [3-6]. The Childbirth Acquired Perineal Trauma (CHAPTER) cohort study is designed to address this substantial evidence gap.

This multicentre, prospective, UK-based cohort study will follow a diverse group of UK women who have sustained CRPT. We will determine the current assessment and management of CRPT immediately after it is sustained, in addition to the prevalence and type of short- and long-term complications in women who have CRPT. Risk factors for developing CRPT complications and current practice of assessment and management of CRPT complications will be ascertained.

Methods and analysis

Objectives

• To describe the current assessment and management of CRPT at the time it is sustained

• To determine the prevalence of short- and long-term complications or health problems in women who have CRPT

• To describe the management of subsequent complications in women with CRPT

• To describe the risk factors associated with the development of CRPT associated complications or longer-term sequalae

For peer review only - http://bmjopen.bmj.com/site/about/guidelines.xhtml

Study design

A UK multi-centre prospective cohort study

Study setting

CHAPTER is open to any type of unit in the UK which offers maternity care. This includes obstetric-led, alongside midwifery units and freestanding midwifery units.

Sample size calculations

We will aim to recruit a minimum of 1000 women. 300 participants will allow us to determine a CRPT complication prevalence rate of 10% with a 95% confidence interval (CI) of 6.8%-14.0%. A sample size of 900 will allow rarer outcomes to be estimated with precision, for example a 95% CI for an estimate of 5% would range from 3.7% to 6.6%. We anticipate the sample will include approximately 450 women in the minor trauma group and 450 women in the second, third and fourth degree tear group. We have in excess of 99% power to detect the difference in pain relief previously seen and with the anticipated sample we would be able to detect a difference of less than 10 percentage points between groups (25% vs 35%).

Approximately 70% of women have a vaginal delivery (55-60% unassisted; 10-15% assisted) and there are 73,889 live births per six week period, thus 51,722 with a vaginal delivery of which 39,723 will sustain non-obstetric anal sphincter injury (non-OASI) CPRT and 2,068 OASI CRPT [1, 10]. Based on 20 maternity sites participating (n=9,930) and 80% of those being suitable to be approached (eligible, baby or mother not too unwell to be approached – 7,944), if 50% of those eligible are successfully approached with 50% consenting (1,986) and 50% completing all follow-up gives a potential sample of n=993 for all outcomes. For those outcomes that can be determined by routine data collection this gives a potential sample of n=1,986 [11, 12].

For peer review only - http://bmjopen.bmj.com/site/about/guidelines.xhtml

Inclusion criteria

To include women who fit any of the following criteria:

- women (≥ 16 yrs) who have given birth either spontaneously or operatively and sustained CRPT (including OASI) who are able and willing to give informed consent
    - to include women who have failed instrumental delivery who ultimately give birth via caesarean section provided they have sustained CRPT
    - to include women with multiple births who give birth vaginally and via caesarean section provided they have sustained CRPT

For peer review only

Exclusion criteria

None

Outcomes to be measured

Primary outcome: perineal infection within six weeks post-birth

Secondary outcomes:

Clinical outcomes from medical records check

- Antibiotic use for perineal infection
- Wound breakdown
- Urinary and faecal incontinence
- Readmission or triage visit for CRPT-related complications
- Requiring referral or review within a specialist perineal clinic
- Requiring minor or major corrective perineal surgery

Patient-reported outcomes as obtained from questionnaires

At six-weeks, six and 12 months post-partum:

- Urinary incontinence via the Revised Urinary Incontinence Scale (RUIS) [13]

For peer review only - http://bmjopen.bmj.com/site/about/guidelines.xhtml

- Faecal incontinence via the Revised Faecal Incontinence Scale (RFIS) [14]

- Anxiety and depression via the Edinburgh Postnatal Depression Scale [15]

- General health related quality of life using the EQ-5D-5L [16]

- Post-traumatic stress disorder (PTSD) via selected domains from the City Birth Trauma Scale [17]

- Measures of pain

- Readmissions

At six and 12-months post-partum only:

- Sexual function via the Bespoke CHAPTER Questionnaire

- Physical activity via the Pelvic Floor Impact Questionnaire [18]

- Pelvic organ prolapse symptoms via selected domains from the Revised Postpartum Pelvic Floor and Birth Questionnaire [19]

- Maternal satisfaction

- Ability to feed baby according to mother's preference

- Ability to care for baby

- Social isolation

- Ability to care for older children

- Impact on seeking healthcare

- Future pregnancy and subsequent mode of birth preference

Identification of participants and recruitment

Potentially eligible participants will be identified by members of the clinical care or research team at the relevant site. This will include midwives and obstetricians (consultants and trainees), research midwives and nurses in maternity units in secondary and tertiary care NHS Trusts. All participants screened for inclusion in the CHAPTER study will be recorded on the online CHAPTER screening log. No identifiable information will be included on the screening log. This log will be prospectively maintained at each site using either the hospital birth register or the postnatal ward-specific list of

For peer review only - http://bmjopen.bmj.com/site/about/guidelines.xhtml

For peer review only

births. The CHAPTER Screening Log will be held on the Research Electronic Data Capture (REDCap) system.

There will be a maximum of three defined 14-day data collection periods. We shall review demographics and type of CRPT after each 14-day data collection period to ensure that we are achieving representation related to non-OASI and our aim for inclusivity. This will allow us to purposively recruit in subsequent rounds if required.

Consent process

Within the CHAPTER Cohort Study, two different methods can be used to obtain informed consent for the study:

- In-person written informed consent
- Remote consent

Irrespective of the type of consent to be used, the first approach to potential participants for possible inclusion in the study must be made by a member of the clinical care team at site (e.g. midwife or obstetrician).

The initial approach to potential participants will, in most cases, take place while women are still an inpatient and can be in one of two settings:

- On the labour ward/birthing unit for those women anticipating an early discharge but at least six hours after birth.
- On the post-natal ward.
- For those women who have a home birth or who are discharged before they have been approached for inclusion, they will be asked to participate when they are seen by a community midwife usually at day one post-partum.

For peer review only - http://bmjopen.bmj.com/site/about/guidelines.xhtml

Women will be provided with background information about the study, including the premise and what their participation would involve. The women will have an opportunity to ask questions and a written CHAPTER study patient information sheet (PIS) will be provided as an adjunct to this conversation.

Those for whom English is not their first spoken language will also be supported by standard Trust interpretation services, for example Trust interpreters or Language Line (a UK language translation service agency that provides interpreting and translation services in over 200 languages, including British Sign Language). Women who understand spoken English will be able to participate in the study. They will be contacted via telephone and asked to complete the follow-up questionnaires over the telephone with a translator should they choose to. A record of the discussion with the women will be kept in their medical notes.

Remote consent process

Remote documented consent is an option within the CHAPTER Study. Remote consent may be undertaken if in-person consent is not feasible, for example in areas where participants are likely to have a homebirth due to the geographical area. In these instances, the woman will be provided with the PIS and consent form by the community midwife or by post. The informed consent discussions will proceed as detailed, but by telephone or video-conference and the details will be recorded in the patient's medical notes.

There are then two options which may be used to document informed consent. The choice of which method is the decision of the research team at site:

- Scanned/ photograph of consent form:

The patient will be asked to initial the boxes on the consent form, and sign and date the form whilst on the call. If the consent form was sent to the patient by post the patient will be asked to take a photo or scan of the signed form, and email it to the site staff, who will print it off and counter-sign. If the consent form was sent to the patient electronically, and they have access to a printer, they can print a

For peer review only

For peer review only - http://bmjopen.bmj.com/site/about/guidelines.xhtml

hard copy, take a photo or scan of the signed form, and email it to the site staff, who will print it off and counter-sign. The original signed and counter-signed consent form will be placed in the Investigator Site File (ISF), a copy will be sent to the participant (electronically or by post), and a copy will be filed in the patient's medical notes.

-Postal consent:

The patient will be asked to initial the boxes on the consent form, and sign and date the form whilst on the call. The patient should then post the signed consent form to the site staff, who will counter-sign it upon receipt. The original signed and counter-signed consent form will be placed in the ISF, a copy will be posted to the participant, and a copy will be filed in the patient's medical notes.

Withdrawal of consent

Participants will be made aware from the beginning of their involvement in the study that they can freely withdraw at any time. Continued consent will be assumed by the completion and return of the questionnaires at each time point. Participants will be given details on how to withdraw from the study at any point in the PIS. Reasons for withdrawal must be recorded on the CHAPTER REDCap database.

If a participant decides that they do not wish to continue their participation in the CHAPTER Study, they may withdraw in one of two ways:

1) No study related follow-up: The participant does not wish to complete patient-reported study-specific follow-up (i.e. patient reported outcomes and quality of life measures at six weeks, six- and 12-months postpartum) but is willing for routinely collected data to be used for inclusion in the study.

2) No further data collection: The participant is not willing to be followed up in any way for the purposes of the study AND does not wish for any further data to be collected (i.e., only data collected prior to withdrawal can be used in the study analysis).

For peer review only - http://bmjopen.bmj.com/site/about/guidelines.xhtml

For peer review only

If a participant withdraws from the CHAPTER Study, their study record on the CHAPTER REDCap database will be closed and no further data will be collected, as per the participant's request. All data collected until that time-point, will be retained and included in the analysis.

Participant Registration

Registration will be via a secure online system hosted by the University of Birmingham Centre for Prospective and Observational Studies (BiCOPS); www.CHAPTER-bicops.bham.ac.uk. Unique log-in usernames will be provided to those who wish to use the online system and who have been delegated the role of registering participants into the study.

After eligibility for registration has been confirmed and informed consent has been taken and documented, the participant can be registered to the study using the CHAPTER online system. During the online registration process, eligibility must be confirmed and participant contact details for follow-up must be entered. A Registration Number will be given once all questions have been answered. Following registration, a confirmatory e-mail will be sent to the responsible clinician, midwife and local Principle Investigator (PI). The local research team should add the participant to the CHAPTER participant recruitment and identification log which links participants with their CHAPTER study number. PIs must maintain this document securely and it must not be submitted to the study office. The CHAPTER participant recruitment and identification log should be held in strict confidence.

Once the patient has consented for entry into CHAPTER, their GP should be notified that they are in the CHAPTER study, using the CHAPTER study GP Letter. No other parties outside of the study team will be informed of the participant's entry into the study.

Baseline data collection

After the woman has consented and they are registered on to the CHAPTER study, baseline information will be collected (outline of information collected in table 1). Information will be collected

For peer review only - http://bmjopen.bmj.com/site/about/guidelines.xhtml

from the maternity record related to demographics (e.g. age, ethnicity), maternal characteristics (e.g. body mass index, obstetric history, medical conditions) and delivery characteristics (e.g. mode of delivery, CRPT type and details of repair). There are no additional study-specific follow-up hospital visits once the participant is entered into the CHAPTER study.

Table 1: A summary of baseline study participant characteristics

Category	Baseline data item
Demographic information	Age
	Ethnicity
	Geographical location
Maternal characteristics	Booking weight
	Body mass index
	Comprehensive obstetric history including previous tear details
	Medical conditions pre-existing or during pregnancy
	Smoking status
	Female genital mutilation status, type and deinfibulation
Labour and birth details	Onset of labour: spontaneous or induced
	Length of second stage
	Singleton/multiples birthed
	Mode and setting of delivery
	Presentation and position at birth
	Birth outcome: livebirth/stillbirth
	Episiotomy
	Hands on/poised/off perineum at crowning
	Maternal position
	Shoulder dystocia and management if occurred
	Blood loss
	Analgesia used in labour
	Presence of chorioamnionitis
	Use of antibiotics in labour or immediately postpartum and indication
	Baby details: gestation, sex, weight and measurements
Tear details, repair details	Grade and role of clinician who inspected perineum after birth
	Classification of tear: first degree tear, second degree tear, third degree tear, fourth degree tear, episiotomy, other lacerations
	Repair performed: method, suture material, by whom, in which setting
	Analgesia during and after repair
	Examinations performed before/after repair (vaginal examination/per rectal examination) and outcome
	Did baby stay with mother during repair?
	Was follow-up of tear arranged?

Data collection timepoints

All follow-up will be conducted remotely. At the time of study entry, participants will be asked to specify their preferred method of contact. Participants will be contacted at the follow-up timepoints, using their

For peer review only - http://bmjopen.bmj.com/site/about/guidelines.xhtml

preferred method of communication where possible to request completion of the CHAPTER study questionnaire (S1 Appendix— six-week questionnaire). There are three follow-up timepoints at six weeks, six months and 12 months postpartum.

At six weeks postpartum: A clinical note review of electronic secondary care records will be undertaken. This combined with the questionnaire data at this time-point, will be used to determine whether the primary outcome of perineal infection within six weeks post-partum has been met.

At all time-points, participants will be asked to complete questionnaire items relating to:
urinary incontinence, flatus incontinence, faecal incontinence, anxiety and depressive symptoms, general health related quality of life, PTSD symptoms (please see outcomes section for specific questionnaires used).

At six and 12 months postpartum only: participants will be asked questions relating to sexual function, physical activity, pelvic organ prolapse, maternal satisfaction, ability to feed the baby according to mother's preference, ability to care for baby, social isolation, ability to care for older children (if relevant), impact on seeking healthcare, future pregnancy and mode of birth preference.

Where questionnaires indicate that a woman had to seek medical care related to her CRPT, further data will be collected by reviewing the secondary care medical records.

Independent data validators will assess a proportion of the data from included to assess generalisability, inclusivity and data accuracy via validation of the primary outcome and critical data items.

Safety considerations and assessment of risk

CHAPTER is a cohort study, therefore any interventions that CHAPTER participants undergo will be as part of their routine clinical care. For this reason, there are no safety considerations relating to the

For peer review only - http://bmjopen.bmj.com/site/about/guidelines.xhtml

For peer review only

study. We do not anticipate any risks to study participants. Enrolment in the study will not have any impact on patient care as participants will be receiving the same treatment that they would if they were not enrolled. There is a potential benefit for participants in that contact with a member of the research team may identify a woman experiencing concerning symptoms and/or signs of a physical or mental illness. Any women identified through questionnaires as being particularly distressed will be contacted and offered support by the research team with signposting to appropriate local services. Safeguarding elements related to significant concerns related to physical or mental health will involve contact with local services (primary and/or secondary care as appropriate).

Study participants may find the follow-up contacts for completion of the questionnaires an inconvenience given that the follow-up period continues up to 12 months post study entry. We will mitigate any potential risk of attrition bias by ensuring that participants fully understand the time commitment at enrolment stage and that they know they may withdraw at any time. In accordance with the University of Birmingham standard operating procedures, this study has been risk assessed to clarify any risks relating uniquely to this study beyond that associated with usual care. A risk assessment has been conducted and concluded that this study presents no higher risk than the risk of standard medical care.

Women and clinicians may become distressed when completing the questionnaires. We have therefore developed care pathways within the study to manage this (S2 Appendix) [20]. For participants who have completed the questionnaires electronically we will ask them if they have found the questionnaires distressing and would like additional support from the CHAPTER team. Should participants indicate distress and would like a telephone call, they will be contacted within seven days and a member of the team will direct them towards appropriate postnatal support services and their GP. If a women experiences distress while completing the questionnaires with the clinician over the telephone she will be offered an opportunity to stop the questionnaire, or postpone to a later point and directed to support services as above. Should the clinicians conducting the telephone questionnaires, who are trained midwives/obstetricians experienced in the care of postnatal women with perineal

For peer review only

For peer review only - http://bmjopen.bmj.com/site/about/guidelines.xhtml

trauma, become distressed by women's experiences and/or symptoms, they will be offered support within the CHAPTER team though the chief investigator, in keeping with the normal clinical pathways for doctors and midwives working in the NHS.

Data management

Data entry will be completed by the sites via a bespoke BiCOPS REDCaP study database. The data capture system will conduct automatic range checks for specific data values to ensure high levels of data quality. Queries and requests for missing clinical data will be raised using data clarification forms via the study database, with the expectation that these queries will be completed by the site within 30 days of receipt.

Participant questionnaires will be completed online, by post or telephone according to participant preference. At the time of consent and entry into CHAPTER, what the study involves will be discussed with each woman. It will be explained that completion of quality-of-life questionnaires plus other patient-reported outcomes will be requested until 12 months postpartum and the methods of completion of the questionnaires discussed. Participants who have not returned completed forms will be contacted up to three times after the due date to request completion of the questionnaires. After this time, unreturned forms will be regarded as missing and will not be requested again. At the six week time-point, participant questionnaires can be completed at six weeks postpartum, +/- one week. At the six and 12 month time-points, participant questionnaires can be completed +/- one month.

University of Birmingham has policies in place, which are designed to protect the security, accuracy, integrity and confidentiality of personal data. The study is registered with the Data Protection Officer at the University of Birmingham and will hold data in accordance with the Data Protection Act (2018 and subsequent amendments). The CHAPTER Study Office has arrangements in place for the secure storage and processing of the study data which comply with University of Birmingham policies.

For peer review only - http://bmjopen.bmj.com/site/about/guidelines.xhtml

It is the responsibility of the PI to ensure all essential study documentation and source documents (e.g., signed consent forms, Investigator Site Files, participants' hospital notes) at their site are securely retained for the contractual period. Archiving will be authorised by BiCOPS following submission of the end of study report. No documents should be destroyed without prior approval from the BiCOPS Director. The electronic study master file will be stored at BiCOPS for at least three years after the end of the study. Long-term offsite data archiving facilities will be considered for storage after this time; data will be stored securely and confidentially for at least 25 years.

End of study definition

The end of study will be when the last enrolled participant completes their 12-month questionnaires, all database queries have been resolved and the database has been locked and analyses completed. The CHAPTER Study Office will notify the Research Ethics Council (REC) and the Sponsor within 90 days of the end of study. The CHAPTER Study Office will provide the REC and the Sponsor with a summary report of the study.

Data analyses and statistical considerations

Summary statistics: Simple descriptive statistics will be used to summarize characteristics and outcomes, with categorical variables expressed as percentages and continuous variables as mean averages alongside the corresponding standard deviation. To allow for hospital variation, differences in primary and secondary outcomes by participant characteristics will be assessed using multilevel logistic and linear regression models; unadjusted and adjusted models, using clinically plausible explanatory variables, will be reported. Further analysis will investigate potential sub-groups, identified from our ongoing systematic reviews.

Explanatory variables/characteristics: We plan to explore the risk of our outcomes adjusted for variables deemed potentially significant. These will be identified in the following ways: other work packages within the programme grant related to primary care date and systematic reviews of existing literature, patient and public involvement and clinical co-applicants.

For peer review only - http://bmjopen.bmj.com/site/about/guidelines.xhtml

Status and timeline of study

The CHAPTER cohort study started participant recruitment on 1st September 2023 and has undergone two rounds of recruitment to-date (correct as of 19th March 2024). Questionnaires have followed at the initial six month time-point and are currently being distributed at the six month time-point. The 12 month questionnaires will follow accordingly from 1st September 2024 onwards.

Data availability

Requests for data generated during this study will be considered by BiCOPS. Data will typically be available six months after the primary publication. Only scientifically sound proposals from appropriately qualified Research Groups will be considered for data sharing. The request will be reviewed by the BiCOPS Data Sharing Committee in discussion with the CI and, where appropriate (or in absence of the CI) any of the following: the study sponsor, the study management group, and the programme steering committee. A formal Data Sharing Agreement (DSA) may be required between respective organisations once release of the data is approved and before data can be released. Data will be fully de-identified (anonymised) unless the DSA covers transfer of participant identifiable information.

Patient and public involvement statement

Patient and public involvement has been key to the CHAPTER project. We have a patient advisory group (PAG) who have been providing guidance on the development and initiation of this cohort study. The PAG have been instrumental in developing study materials, ethical considerations and in guiding decisions about rounds of recruitment needed. The PAG will continue to guide all aspects of the CHAPTER project development.

For peer review only - http://bmjopen.bmj.com/site/about/guidelines.xhtml

For peer review only

Ethics and dissemination

Ethical approval has been granted by the Research Ethics Council with reference 23/WA/0169. It is anticipated that minor amendments may need to be submitted after commencement of the study. These will be dealt with via submission to the ethics board and subsequently communicating this amendment to sites to ensure their consent is in-place.

Results of the study will ultimately be submitted for publication in a peer reviewed journal after study completion. Findings will also be submitted to national and international conferences as well as disseminated to the Department of Health and Royal Colleges. Appropriate resources for patients will be developed and any relevant findings are expected to be rapidly incorporated into guidelines by the Royal College of Obstetricians and Gynaecologists, the Royal College of Midwives and NICE.

Word count 3,976

Acknowledgments

This project is part of the CHAPTER programme grant and therefore a larger group of collaborators have contributed to the project and are detailed as part of the CHAPTER group.

Competing interests statement

None known

For peer review only - http://bmjopen.bmj.com/site/about/guidelines.xhtml

Funding statement

This project is funded by the National Institute for Health and Care Research (NIHR), grant number NIHR202869. The views expressed are those of the authors and not necessarily those of NIHR or the Department of Health and Social Care.

Author contributions

VHM- initial protocol development, design of methodology, overseeing protocol revisions, RM- writing protocol for submission, editing and revisions, RP- project administration, TH- data curation, project administration, ST- project administration, CM- conceptualization, design of methodology, drafting protocol, LM- conceptualization, design of methodology, RKM- initial protocol development, conceptualization, funding acquisition, design of methodology. CHAPTER group: conceptualization, funding acquisition, methodology. All authors read and approved the final manuscript.

References

1. NHS Digital. NHS Maternity Statistics 2018-2019 [online]. 2019. https://files.digital.nhs.uk/D0/C26F84/hosp-epis-stat-mat-summary-report-2018-19.pdf (accessed 10 August 2023).

2. Smith LA, Price N, Simonite V, et al. Incidence of and risk factors for perineal trauma: a prospective observational study. BMC Pregnancy Childbirth. 2013;13(1): 59. doi: 10.1186/1471-2393-13-59

3. Andrews V, Thakar R, Sultan AH, et al. Evaluation of postpartum perineal pain and dyspareunia—a prospective study. Eur J Obstet Gynecol Reprod Biol. 2008;137(2):152-156.

4. Leeman L, Rogers R, Borders N, et al. The effect of perineal lacerations on pelvic floor function and anatomy at 6 months postpartum in a prospective cohort of nulliparous women. Birth. 2016;43(4): 293-302.

For peer review only - http://bmjopen.bmj.com/site/about/guidelines.xhtml

5. Dudley L, Kettle C, Ismail K. Prevalence, pathophysiology and current management of dehisced perineal wounds following childbirth. Br J Midwifery. 2013;21(3):160-171.

6. McDonald E, Gartland D, Small R, et al. Dyspareunia and childbirth: a prospective cohort study. BJOG. 2015;122(5): 672-679.

7. Opondo C, Harrison S, Sanders J, et al. The relationship between perineal trauma and postpartum psychological outcomes: a secondary analysis of a population-based survey. BMC Pregnancy Childbirth. 2023;23(1): 639. doi: 10.1186/s12884-023-05950-6.

8. Priddis H, Schmied V, Dahlen H. Women's experiences following severe perineal trauma: a qualitative study. BMC Womens Health. 2014;14(1): 32. doi: 10.1186/1472-6874-14-32.

9. National Institute for Health and Clinical Excellence. Postnatal Care [online]. 2021. https://www.nice.org.uk/guidance/ng194 (accessed 11 August 2023).

10. NMPA Project Team. National maternity and perinatal audit: clinical report 2019 [online]. 2019. https://www.hqip.org.uk/resource/national-maternity-and-perinatal-audit-nmpa-clinical-report-2019/ (accessed 1 March 2024).

11. Knight M, Chiocchia V, Partlett C, et al. Prophylactic antibiotics in the prevention of infection after operative vaginal delivery (ANODE): a multicentre randomised controlled trial. Lancet. 2019;393(10189): 2395-2403.

12. Hodgetts-Morton V, Hewitt CA, Wilson A, et al. Vaginal preparation with chlorhexidine at cesarean section to reduce endometritis and prevent sepsis: A randomized pilot trial (PREPS). Acta Obstet Gynecol Scand. 2020;99(2): 231-239.

13. Sansoni JE, Marosszeky N, Sansoni E, et al. The development of the revised urinary incontinence scale (RUIS): Australian health outcomes collaboration [online]. 2008. https://ro.uow.edu.au/gsbpapers/64 (accessed 22 February 2024).

14. Sansoni J, Hawthorne G, Fleming G, et al. The revised faecal incontinence scale: a clinical validation of a new, short measure for assessment and outcomes evaluation. Dis Colon Rectum. 2013;56(5): 652-659.

15. Cox JL, Holden JM, Sagovsky R. Detection of postnatal depression: development of the 10-item edinburgh postnatal depression scale. Br J Psychiatry. 1987;150(6): 782-786.

For peer review only

For peer review only - http://bmjopen.bmj.com/site/about/guidelines.xhtml

16. Herdman M, Gudex C, Lloyd A, et al. Development and preliminary testing of the new five-level version of EQ-5D (EQ-5D-5L). Qual Life Res. 2011;20(10): 1727-1736.

17. Ayers S, Wright DB, Thornton A. Development of a measure of postpartum PTSD: the city birth trauma scale. Front Psychiatry. 2018;9. doi: 10.3389/fpsyt.2018.00409.

18. Barber MD, Kuchibhatla MN, Pieper CF, et al. Psychometric evaluation of 2 comprehensive condition-specific quality of life instruments for women with pelvic floor disorders. Am J Obstet Gynecol. 2001;185(6): 1388-1395.

19. Thibault-Gagnon S, Yusuf S, Langer S, et al. Do women notice the impact of childbirth-related levator trauma on pelvic floor and sexual function? Results of an observational ultrasound study. Int Urogynecol J. 2014;25(10): 1389-1398.

20. Draucker CB, Martsolf DS, Poole C. Developing distress protocols for research on sensitive topics. Arch Psychiatr Nurs. 2009;23(5): 343-350.

Supporting information captions :

S1 Appendix- 6 week questionnaire

S2 Appendix- distress pathway

For peer review only

For peer review only - http://bmjopen.bmj.com/site/about/guidelines.xhtml

S1 Appendix: Six week Quality of life questionnaire

Date added to REDCap 6 week QoL

____

6 Week QoL Questionnaire

You kindly agreed to take part in the Chapter cohort study following the birth of your baby. This research aims to understand how common complications are amongst women who have experienced childbirth acquired perineal trauma. To do this, we would like to collect some information from you at 6 weeks, 6 months and 12 months following the birth of your baby.

We have used three words frequently in the questionnaire - perineum, stitches and tear. Perineum is the part of your body between the opening of your vagina and your back passage. Stitches and tear refer to the cuts or tears in your perineum or vaginal area (either made by the midwife/doctor or happening on their own) during the birth of your baby.The perineum connects with the muscles in the lower pelvis to form the pelvic floor. Your pelvic floor helps to control your bladder, bowel and sexual function.

We expect the questionnaire will take you approximately 20 minutes to complete. It is divided into 4 sections:

Section 1: This section asks in detail about the healing of your stitches/tear and how satisfied you felt about the support you received from healthcare professionals in relation to the healing of your perineum. It asks whether you have had an infection or any complications and whether you have needed to be readmitted to hospital for treatment.

Section 2: This section explores if any symptoms related to the healing of your stitches/tear may have impacted the experience of feeding and caring for your baby (and older children, if applicable).

Section 3: This section will focus on specific questions relating to whether there have been any changes to your pelvic floor and how this impacts your bladder and bowel function. It also asks how these changes influence your day-to-day life.

Section 4: The last part of the questionnaire asks several questions about your mental health and wellbeing.

Within the Chapter study we use the term woman to refer to all birthing people as we acknowledge that it is not only people who identify as women who may be affected by childbirth related perineal trauma.

Sharing your experiences with us will increase our knowledge and lead to better understanding in this area. All the information that you provide will be confidential and may help us to improve the care women receive following childbirth.

By completing this questionnaire, you are confirming that you are still happy to take part in this research. If you decide you wish to withdraw from this study or have any queries, please contact the Chapter study research team on chapter@contacts.bham.ac.uk.

If at any time you feel this questionnaire is distressing you. you can use the 'save & return later' button to leave. If you wish you can return and complete the rest of the questionnaire at a more convenient time.

Thank you again for your participation and please remember that we are more than happy for you to contact us if you need any assistance with completing the questionnaire.

For peer review only

Section 1: This section asks in detail about the healing of your stitches/tear and how satisfied you felt about the support you received from healthcare professionals in relation to the healing of your perineum. It asks whether you have had an infection or any complications and whether you have needed to be readmitted to hospital for treatment.

Have you had any problems with the healing of your stitches or tear?

◯ Yes ◯ No

Since the birth of your baby have you noticed any discharge or leakage of fluid from any part of the stitches/tear?

◯ Yes ◯ No

Was the discharge or leakage either:

◯ Clear or blood stained
◯ Yellow/ green (pus)
◯ Other,please specify

Describe discharge or leakage:

____

Please tick any of the following additional symptoms that apply to your stitches/ tear: ______

______

When did you first notice any of these symptoms?

◯ 0-2 weeks after having my baby
◯ 2-4 weeks after having my baby
◯ 4-6 weeks after having my baby
◯ Unsure

Please check you have not missed any questions above before you continue to the next section. If you do not want to answer any questions for any personal reasons, that is fine.

Did you see a health care professional for any problems connected with your stitches or tear during this time?

◯ Yes ◯ No

Please indicate which health care professional you saw in the period following the birth of your baby for any problems connected with your stitches/tear

Doctor in hospital

◯ Yes ◯ No

How satisfied you were with the support you were given in relation to the healing of your stitches/tear from the Doctor at hospital

◯ Very satisfied
◯ Satisfied
◯ Unsatisfied
◯ Very unsatisfied

For peer review only

How satisfied you were with the support you were given in relation to the healing of your stitches/tear from the Doctor at hospital

◯ Very satisfied
◯ Satisfied
◯ Neutral
◯ Unsatisfied
◯ Very unsatisfied

Midwife at hospital

◯ Yes ◯ No

How satisfied you were with the support you were given in relation to the healing of your stitches/tear from the Midwife

◯ Very satisfied
◯ Satisfied
◯ Unsatisfied
◯ Very unsatisfied

How satisfied you were with the support you were given in relation to the healing of your stitches/tear from the Midwife

◯ Very satisfied
◯ Satisfied
◯ Neutral
◯ Unsatisfied
◯ Very unsatisfied

Midwife in community

◯ Yes ◯ No

How satisfied you were with the support you were given in relation to the healing of your stitches/tear from the Midwife

◯ Very satisfied
◯ Satisfied
◯ Unsatisfied
◯ Very unsatisfied

How satisfied you were with the support you were given in relation to the healing of your stitches/tear from the Midwife

◯ Very satisfied
◯ Satisfied
◯ Neutral
◯ Unsatisfied
◯ Very unsatisfied

Your General Practioner (GP)

◯ Yes ◯ No

For peer review only

How satisfied you were with the support you were given in relation to the healing of your stitches/tear from the GP

◯ Very satisfied
◯ Satisfied
◯ Unsatisfied
◯ Very unsatisfied

How satisfied you were with the support you were given in relation to the healing of your stitches/tear from the GP

◯ Very satisfied
◯ Satisfied
◯ Neutral
◯ Unsatisfied
◯ Very unsatisfied

Health visitor

◯ Yes ◯ No

How satisfied you were with the support you were given in relation to the healing of your stitches/tear from the Health visitor

◯ Very satisfied
◯ Satisfied
◯ Unsatisfied
◯ Very unsatisfied

How satisfied you were with the support you were given in relation to the healing of your stitches/tear from the Health visitor

◯ Very satisfied
◯ Satisfied
◯ Neutral
◯ Unsatisfied
◯ Very unsatisfied

Physiotherapist at the hospital

◯ Yes ◯ No

How ssatisfied you were with the support you were given in relation to the healing of your stitches/tear from the Physiotherapist

◯ Very satisfied
◯ Satisfied
◯ Unsatisfied
◯ Very unsatisfied

How ssatisfied you were with the support you were given in relation to the healing of your stitches/tear from the Physiotherapist

◯ Very satisfied
◯ Satisfied
◯ Neutral
◯ Unsatisfied
◯ Very unsatisfied

For peer review only

For peer review only - http://bmjopen.bmj.com/site/about/guidelines.xhtml

Private physiotherapy

◯ Yes ◯ No

How satisfied you were with the support you were given in relation to the healing of your stitches/tear from the private Physiotherapist

◯ Very satisfied
◯ Satisfied
◯ Unsatisfied
◯ Very unsatisfied

How satisfied you were with the support you were given in relation to the healing of your stitches/tear from the private Physiotherapist

◯ Very satisfied
◯ Satisfied
◯ Neutral
◯ Unsatisfied
◯ Very unsatisfied

Other heathcare professional

◯ Yes ◯ No

Confirm which other healthcare professional you have received advice and support from:

____

How satisfied you were with the support you were given in relation to the healing of your stitches/tear from the [anode_hcw_other_name]

◯ Very satisfied
◯ Satisfied
◯ Unsatisfied
◯ Very unsatisfied

How satisfied you were with the support you were given in relation to the healing of your stitches/tear from the [anode_hcw_other_name]

◯ Very satisfied
◯ Satisfied
◯ Neutral
◯ Unsatisfied
◯ Very unsatisfied

You have indicated that you didn't seek any further assistance from a health care professional in relation to your stitches/tear. Please indicate why you did not seek assistance. You can tick more than one - please tick ALL that apply.

☐ I didn't feel I needed to as I recovered well after the birth
☐ I wasn't aware of any services that could help me
☐ I was too embarrassed to discuss my symptoms and concerns
☐ I was fearful about an intimate examination
☐ I had some concerns and knew services existed but I haven't been able to find the time to seek further assistance
☐ Other

PLease specify Other reason you did not seek assistance.

____

For peer review only
For peer review only - http://bmjopen.bmj.com/site/about/guidelines.xhtml

Please check you have not missed any questions above before you continue to the next section. If you do not want to answer any questions for any personal reasons, that is fine.

Hospital Visits

Did you have to go to a hospital clinic as an out-patient for any problems connected with your stitches/tear since the birth of your baby?

◯ Yes ◯ No

What was the reason you attended the hospital as an out-patient? You can tick more than one - please tick ALL that apply.

☐ Pain or soreness in addition to the discomfort experienced following the birth
☐ Redness or inflammation spreading from the edges of the stitches/tear
☐ The area around the stitches/tear felt warmer/hotter than the surrounding skin
☐ The area around the stitches/tear became swollen
☐ The edges of any part of the stitches/tear separated or gaped open
☐ Other, please specify

Confirm the other reason you had to go to out-patients

____

How many times have you had to go to out-patients

____

Please check you have not missed any questions above before you continue to the next section. If you do not want to answer any questions for any personal reasons, that is fine.

Have you been readmitted to hospital for any reason connected with your own health since the birth of your baby? (We don't need to know if you were admitted with your baby because your baby was unwell)

◯ Yes ◯ No

How many times have you been readmitted for any reason connected with your own health since the birth of your baby?

◯ 1 ◯ 2 ◯ 3 ◯ more than 3

Please tell us about your first readmission to hospital by providing the following details.

Name of hospital of 1st re-admission

____

For peer review only

What was the reason for this first readmission? You can tick more than one - please tick ALL that apply.

☐ Pain or soreness in addition to the discomfort experienced following the birth
☐ Redness or inflammation spreading from the edges of the stitches/tear
☐ The area around the stitches/tear felt warmer/hotter than the surrounding skin
☐ The area around the stitches/tear became swollen
☐ The edges of any part of the stitches/tear separated or gaped open
☐ Other, please specify

Please specify Other reason for 1st re-admission

____

How long after you gave birth to your baby were you readmitted to hospital the first time?

◯ Less than 1 week
◯ 1-2 weeks
◯ 2-3 weeks
◯ 3-4 weeks
◯ 4-5 weeks
◯ 5-6 weeks

Did you have an operation when you were readmitted the first time?

◯ Yes ◯ No

Were you in the intensive care unit when you were readmitted the first time?

◯ Yes ◯ No

How many nights did you stay in hospital during this first readmission?

____

Please tell us about your second readmission to hospital by providing the following details.

Name of hospital of 2nd re-admission

____

What was the reason for this second readmission? You can tick more than one - please tick ALL that apply.

☐ Pain or soreness in addition to the discomfort experienced following the birth
☐ Redness or inflammation spreading from the edges of the stitches/tear
☐ The area around the stitches/tear felt warmer/hotter than the surrounding skin
☐ The area around the stitches/tear became swollen
☐ The edges of any part of the stitches/tear separated or gaped open
☐ Other, please specify

Please specify Other reason for 2nd re-admission

____

For peer review only

How long after you gave birth to your baby were you readmitted to hospital the second time?

◯ Less than 1 week
◯ 1-2 weeks
◯ 2-3 weeks
◯ 3-4 weeks
◯ 4-5 weeks
◯ 5-6 weeks

Did you have an operation when you were readmitted the second time?

◯ Yes ◯ No

Were you in the intensive care unit when you were readmitted the first time?

◯ Yes ◯ No

How many nights did you stay in hospital during this second readmission?

____

Please tell us about your third readmission to hospital by providing the following details.

Name of hospital of 3rd re-admission

____

What was the reason for this third readmission? You can tick more than one - please tick ALL that apply.

☐ Pain or soreness in addition to the discomfort experienced following the birth
☐ Redness or inflammation spreading from the edges of the stitches/tear
☐ The area around the stitches/tear felt warmer/hotter than the surrounding skin
☐ The area around the stitches/tear became swollen
☐ The edges of any part of the stitches/tear separated or gaped open
☐ Other, please specify

Please specify Other reason for 3rd re-admission

____

How long after you gave birth to your baby were you readmitted to hospital the third time?

◯ Less than 1 week
◯ 1-2 weeks
◯ 2-3 weeks
◯ 3-4 weeks
◯ 4-5 weeks
◯ 5-6 weeks

Did you have an operation when you were readmitted the third time?

◯ Yes ◯ No

For peer review only

Were you in the intensive care unit when you were readmitted the third time?

◯ Yes ◯ No

How many nights did you stay in hospital during this third readmission?

____

As you have been re-admitted to hospital more than 3 times please write how many ADDITIONAL nights you have stayed

____

Please check you have not missed any questions above before you continue to the next section. If you do not want to answer any questions for any personal reasons, that is fine.

Have you been prescribed any antibiotics since the birth of your baby? (if you were readmitted to hospital include any antibiotics you were given then).

◯ Yes
◯ No
◯ Unknown drug received
◯ Cannot remember if any medication was received

What were they given for?

☐ Pain or soreness in addition to the discomfort experienced following the birth
☐ Redness or inflammation spreading from the edges of the stitches/tear
☐ The area around the stitches/tear felt warmer/hotter than the surrounding skin
☐ The area around the stitches/tear became swollen
☐ The edges of any part of the stitches/tear separated or gaped open
☐ Other, please specify

Other reason drugs were given

____

Who prescribed them?- please tick ALL that apply.

☐ GP
☐ Doctor in maternity unit
☐ Doctor in emergency department
☐ Other

Other prescriber, please specify

____

Were they prescribed more than 24 hours after the birth of your baby?

◯ Yes ◯ No

Please check you have not missed any questions above before you continue to the next section. If you do not want to answer any questions for any personal reasons, that is fine.

For peer review only - http://bmjopen.bmj.com/site/about/guidelines.xhtml

Has your doctor taken any bloods or swabs to send to the lab to check for infection

◯ Yes
◯ No
◯ Don't know

Have you been told you had an infection in your perineum

◯ Yes
◯ No
◯ Unsure

Please tick all signs and symptoms you experienced:

☐ Fever >38 degrees
☐ Perineal pain
☐ Smelly discharge
☐ Redness around the stiches/tear
☐ Swelling
☐ Other

Other signs and symptoms you experienced:

____

Please check you have not missed any questions above before you continue to the next section. If you do not want to answer any questions for any personal reasons, that is fine.

In the past week, have you experienced any pain or unpleasant feeling in your perineum? (the area between your vagina and back passage)

◯ Yes ◯ No

Would you describe the strength of the pain or unpleasant feeling as?

◯ Mild
◯ Moderate
◯ Severe

Is the pain or unpleasant feeling there?

◯ Some of the time
◯ Most of the time
◯ All of the time

In the past week have you needed any tablets to relieve the pain or discomfort in your perineum?

◯ Yes ◯ No

Please check you have not missed any questions above before you continue to the next section. If you do not want to answer any questions for any personal reasons, that is fine.

For peer review only

How well do you feel that your perineum has healed? . (Please tick one box that is the most appropriate statement for the way you feel)

◯ I feel that my perineum has healed well
◯ I feel that my perineum has healed satisfactorily
◯ I feel that my perineum has healed poorly
◯ I feel that my perineum has healed very poorly

Please check you have not missed any questions above before you continue to the next section. If you do not want to answer any questions for any personal reasons, that is fine.

For peer review only

Section 2: This section explores if any symptoms related to the healing of your stitches/tear may have impacted the experience of feeding and caring for your baby (and older children, if applicable).

Have you breastfed your baby at any time since he/she was born

◯ Yes ◯ No

Are you still breastfeeding your baby

◯ Yes ◯ No

If you are no longer breastfeeding your baby: how old was your baby when you stopped breastfeeding? Please specify to the closest week breastfeeding stopped.

◯ Less than 1 week
◯ Between 1-2 weeks
◯ Between 2-3 weeks
◯ Between 3-4 weeks
◯ Between 4-5 weeks
◯ Between 5-6 weeks

Why did you stop breastfeeding You can tick more than one - please tick ALL that apply.

☐ Painful nipples
☐ Engorgement
☐ Blocked milk duct
☐ Thrush
☐ Mastitis
☐ Breast abscess
☐ Not enough milk
☐ Perineum was too uncomfortable or painful whilst feeding my baby
☐ Other

Other reason stopped

____

Has your perineum been too uncomfortable or painful for you to feed your baby

◯ Yes ◯ No

Have problems or worries about your perineum caused you to not be able to care for your baby in the way you'd like to during this time? (Care includes washing/ feeding/ playing/ holding your baby).

◯ Yes
◯ No
◯ I have not had any problems or worries about my perineum

How often do these problems or worries about your perineum interfere with you caring for your baby:

◯ A lot of the time (for example, more than once a day)
◯ Some of the time (for example, at least once a day)
◯ Not very often

For peer review only

Do you feel problems or worries about your perineum have affected bonding with your baby during this time?

◯ Yes
◯ No
◯ I have not had any problems or worries about my perineum

Have problems or worries about your perineum caused you to not be able to care for your older children in the way you'd like?

◯ Yes
◯ No
◯ I don't have any older children

How often do these problems or worries about your perineum interfere with you caring for your older child?

◯ A lot of the time (for example, more than once a day)
◯ Some of the time (for example, at least once a day)
◯ Not very often

Please check you have not missed any questions above before you continue to the next section. If you do not want to answer any questions for any personal reasons, that is fine.

For peer review only

Section 3: This section will focus on specific questions relating to whether there have been any changes to your pelvic floor and how this impacts your bladder and bowel function. It also asks how these changes influence your relationships and day-to-day life

Please consider your experiences during the last 4 weeks for the following.

Have you had any problems with controlling your bowels? (This includes passing of wind (flatus) and stool).

◯ Yes ◯ No

Do you have difficulty controlling wind (flatus)?

◯ Never
◯ Rarely (for example less than once in the past 4 weeks)
◯ Sometimes (for example less than once a week, but once or more in the past 4 weeks)
◯ Often or usually (for example less than once a day but once a week or more)
◯ Always (for example once or more per day or whenever you have a bowel movement)

Do you leak, have accidents or lose control with solid stool

◯ Never
◯ Rarely i.e. less than once in the past four weeks
◯ Sometimes i.e. less than once a week, but once or more in the past four weeks
◯ Often or usually i.e. less than once a day but once a week or more
◯ Always i.e. once or more per day or whenever you have a bowel movement

Do you leak, have accidents or lose control with liquid stool

◯ Never
◯ Rarely i.e. less than once in the past four weeks
◯ Sometimes i.e. less than once a week, but once or more in the past four weeks
◯ Often or usually i.e. less than once a day but once a week or more
◯ Always i.e. once or more per day or whenever you have a bowel movement

Do you leak stool if you don't get to the toilet in time?

◯ Never
◯ Rarely i.e. less than once in the past four weeks
◯ Sometimes i.e. less than once a week, but once or more in the past four weeks
◯ Often or usually i.e. less than once a day but once a week or more
◯ Always i.e. once or more per day or whenever you have a bowel movement

Does stool leak so that you have to change your underwear

◯ Never
◯ Rarely i.e. less than once in the past four weeks
◯ Sometimes i.e. less than once a week, but once or more in the past four weeks
◯ Often or usually i.e. less than once a day but once a week or more
◯ Always i.e. once or more per day or whenever you have a bowel movement

Does bowel or stool leakage cause you to alter your lifestyle

◯ Never
◯ Rarely i.e. less than once in the past four weeks
◯ Sometimes i.e. less than once a week, but once or more in the past four weeks
◯ Often or usually i.e. less than once a day but once a week or more
◯ Always i.e. once or more per day or whenever you have a bowel movement

Please check you have not missed any questions above before you continue to the next section. If you do not want to answer any questions for any personal reasons, that is fine.

For peer review only - http://bmjopen.bmj.com/site/about/guidelines.xhtml

Please consider your experiences during the last 4 weeks for the following.

Have you had any problems with controlling your bladder?

◯ Yes ◯ No

Do you experience, and if so, how much are you bothered by:

Urine leakage related to the feeling of urgency

◯ Not at all
◯ Slightly
◯ Moderately
◯ Greatly

Urine leakage related to physical activity, coughing or sneezing

◯ Not at all
◯ Slightly
◯ Moderately
◯ Greatly

Small amounts of urine leakage (drops)

◯ Not at all
◯ Slightly
◯ Moderately
◯ Greatly

How often do you experience urine leakage

◯ Never
◯ Less than once a month
◯ A few times a month
◯ A few times a week
◯ Every day and/or night

How much urine do you lose each time

◯ None
◯ Drops
◯ Small splashes
◯ More

Please check you have not missed any questions above before you continue to the next section. If you do not want to answer any questions for any personal reasons, that is fine.

Under each heading, please select the ONE box that best describes your health TODAY

MOBILITY

◯ I have no problems in walking about
◯ I have slight problems in walking about
◯ I have moderate problems in walking about
◯ I have severe problems in walking about
◯ I am unable to walk about

For peer review only

SELF-CARE

◯ have no problems washing or dressing myself
◯ I have slight problems washing or dressing myself
◯ I have moderate problems washing or dressing myself
◯ I have severe problems washing or dressing myself
◯ I am unable to wash or dress myself

USUAL ACTIVITIES (e.g. work, study, housework, family or leisure activities)

◯ I have no problems doing my usual activities
◯ I have slight problems doing my usual activities
◯ I have moderate problems doing my usual activities
◯ I have severe problems doing my usual activities
◯ I am unable to do my usual activities

PAIN / DISCOMFORT

◯ I have no pain or discomfort
◯ I have slight pain or discomfort
◯ I have moderate pain or discomfort
◯ I have severe pain or discomfort
◯ I have extreme pain or discomfor

ANXIETY / DEPRESSION

◯ I am not anxious or depressed
◯ I am slightly anxious or depressed
◯ I am moderately anxious or depressed
◯ I am severely anxious or depressed
◯ I am extremely anxious or depressed

We would like to know how good or bad your health is TODAY. This scale is numbered from 0 to 100.
100 means the best health you can imagine.
0 means the worst health you can imagine.
Please click on the scale to indicate how your health is TODAY.

0 - The worst health you can imagine — 50 — 100 - The best health you can imagine

(Place a mark on the scale above)

Please check you have not missed any questions above before you continue to the next section. If you do not want to answer any questions for any personal reasons, that is fine.

For peer review only

For peer review only - http://bmjopen.bmj.com/site/about/guidelines.xhtml

Section 4: The last part of the questionnaire asks several questions about your mental health and wellbeing.

While it is common to feel a little bit low after having a baby, we would like you to know that there are people who can provide support or information if you are struggling to cope with this.

Getting the right help from a health professional early on leads to a faster recovery. Please speak to your GP or health visitor if there is anything that is troubling you and they will be able to support you. You may also find some of the resources at the end of this questionnaire helpful too.

In the below questions please select the answer that comes closest to how you have felt IN THE PAST 7 DAYS, not just how you feel today.

I have been able to laugh and see the funny side of things

◯ As much as I always could
◯ Not quite so much now
◯ Definitely not so much now
◯ Not at all

I have looked forward with enjoyment to things

◯ As much as I ever did
◯ Rather less than I used to
◯ Definitely less than I used to
◯ Hardly at all

I have blamed myself unnecessarily when things went wrong

◯ Yes, most of the time
◯ Yes, some of the time
◯ Not very often
◯ No, never

I have been anxious or worried for no good reason

◯ No, not at all
◯ Hardly ever
◯ Yes, sometimes
◯ Yes, very often

I have felt scared or panicky for no very good reason

◯ Yes, quite a lot
◯ Yes, sometimes
◯ No, not much
◯ No, not at all

Things have been getting on top of me

◯ Yes, most of the time I haven't been able to cope at all
◯ Yes, sometimes I haven't been coping as well as usual
◯ No, most of the time I have coped quite well
◯ No, I have been coping as well as ever

Please check you have not missed any questions above before you continue to the next section. If you do not want to answer any questions for any personal reasons, that is fine.

For peer review only

This following questions asks about your experience during the birth of your most recent baby. It asks about potential traumatic events during (or immediately after) the labour and birth, and whether you are experiencing symptoms that are reported by some women after birth and how often you have experienced these in the last week. Please tick the responses closest to your experience.

Recurrent unwanted memories of the birth (or parts of the birth) that you can't control

- ◯ Not at all
- ◯ Once
- ◯ 2-4 times
- ◯ 5 or more times

Bad dreams or nightmares about the birth (or related to the birth)

- ◯ Not at all
- ◯ Once
- ◯ 2-4 times
- ◯ 5 or more times

Flashbacks to the birth and/or reliving the experience

- ◯ Not at all
- ◯ Once
- ◯ 2-4 times
- ◯ 5 or more times

Getting upset when reminded of the birth

- ◯ Not at all
- ◯ Once
- ◯ 2-4 times
- ◯ 5 or more times

Feeling tense or anxious when reminded of the birth

- ◯ Not at all
- ◯ Once
- ◯ 2-4 times
- ◯ 5 or more times

Trying to avoid things that remind me of the birth (for example, people, places, TV programmes)

- ◯ Not at all
- ◯ Once
- ◯ 2-4 times
- ◯ 5 or more times

Not able to remember details of the birth

- ◯ Not at all
- ◯ Once
- ◯ 2-4 times
- ◯ 5 or more times

For peer review only

Blaming myself or others for what happened during the birth

◯ Not at all
◯ Once
◯ 2-4 times
◯ 5 or more times

Feeling strong negative emotions about the birth (for example, fear, anger, shame)

◯ Not at all
◯ Once
◯ 2-4 times
◯ 5 or more times

When did these symptoms start?

◯ Before the birth
◯ In the first 6 weeks after the birth

Do these symptoms cause you a lot of distress?

◯ Yes
◯ No
◯ Sometimes

How long have these symptoms lasted?

◯ Less than 1 month
◯ 1 to 2 months

Do they prevent you doing things you usually do (for example, socialising, daily activities?)

◯ Yes
◯ No
◯ Sometimes

Could any of these symptoms be due to medication, alcohol, drugs or physical illness?

◯ Yes
◯ No
◯ Sometimes

Please check you have not missed any questions above before you continue to the next section. If you do not want to answer any questions for any personal reasons, that is fine.

You may have found answering some of these questions distressing. The research team can contact you by telephone to offer support or you can contact your GP. Please indicate below whether you would like the research team to contact you.

◯ I would like to receive a telephone call to offer support.
◯ I do not wish to receive a support call.

Additonal Information

Is there anything else you would like to tell us about the healing of your stitches/tear or how you are feeling now? Please feel free to make any comments important to you in this space.

For peer review only

Thank you very much for taking the time to complete this questionnaire. We will be contacting you to ask you to complete the next questionnaire approximately 6 months following the birth of your baby. If you would like to speak to the Chapter research team about any aspect of this study, please contact the team by email chapter@contacts.bham.ac.uk.

Should you need further support or information at any point please speak to your GP or health visitor. You may find the following resources helpful: www.nhs.uk - to find information and advice on health conditions, symptoms and how to get help www.masic.org.uk - a charity which supports women who have sustained severe perineal trauma during childbirth www.mind.org.uk - a charity that provides advice and support to empower anyone experiencing a mental health problem www.pandasfoundation.org.uk - a charity which provides support for every parent who is struggling with their perinatal mental health

For peer review only

S2 Appendix: Distress Pathway

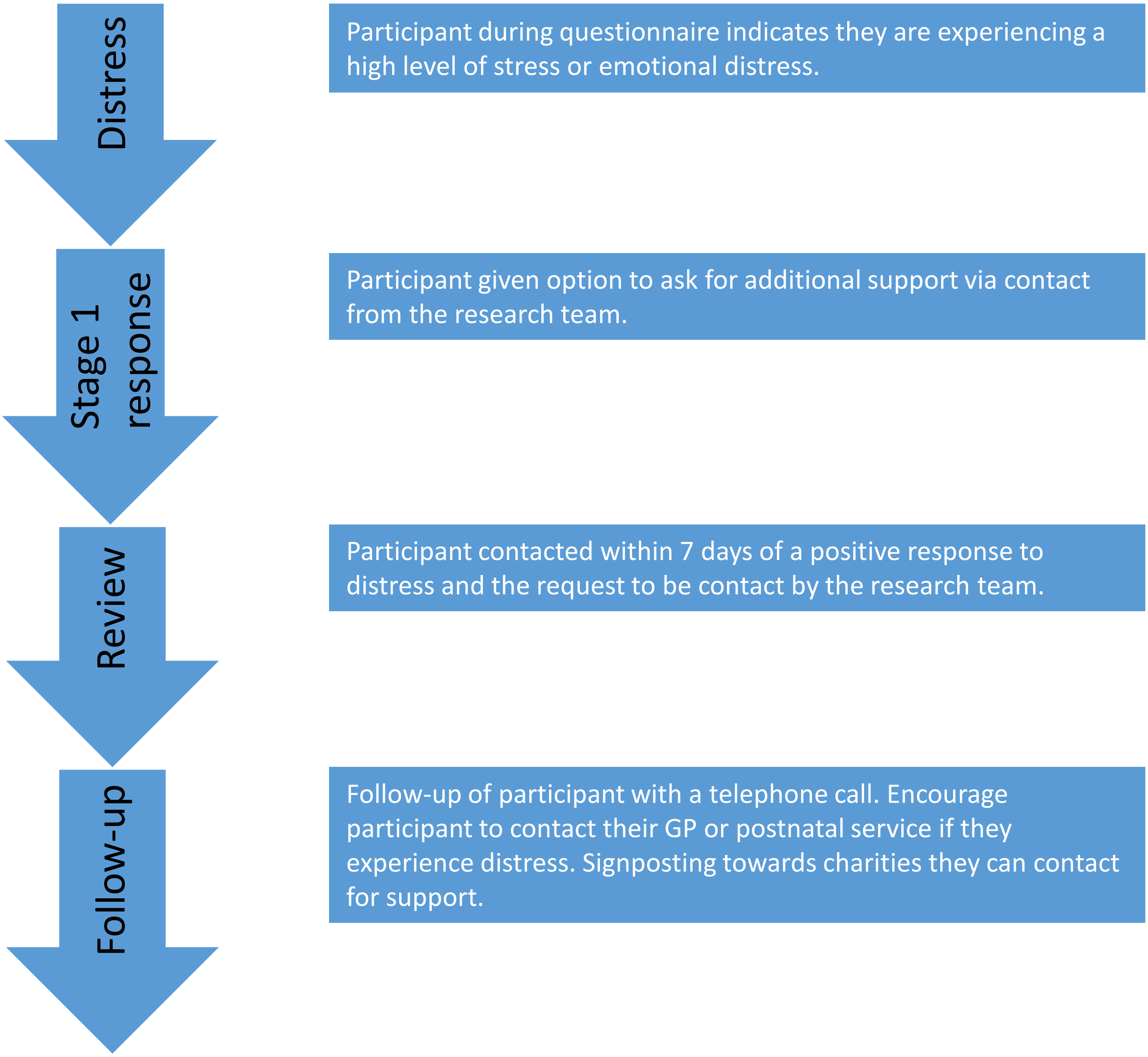

Protocol for managing distress in the context of research on sensitive subjects (adapted from Draucker et al.)
