# Supplementary material for: Childbirth Acquired Perineal Trauma study (CHAPTER): a UK prospective cohort study protocol
Source: BMJ Open. 2024 May 24;14(5):e086724. doi: 10.1136/bmjopen-2024-086724 (PMC11129024; doi:10.1136/bmjopen-2024-086724)
Supplement: Reviewer comments [file bmjopen-2024-086724.reviewer_comments.pdf]

PEER REVIEW HISTORY

BMJ Open publishes all reviews undertaken for accepted manuscripts. Reviewers are asked to complete a checklist review form (**http://bmjopen.bmj.com/site/about/resources/checklist.pdf**) and are provided with free text boxes to elaborate on their assessment. These free text comments are reproduced below.

ARTICLE DETAILS

**TITLE (PROVISIONAL)**	CHILDBIRTH ACQUIRED PERINEAL TRAUMA STUDY (CHAPTER): A UK PROSPECTIVE COHORT STUDY PROTOCOL
**AUTHORS**	Hodgetts Morton, Victoria; Man, Rebecca; Perry, Rita; Hughes, Terry; Tohill, Susan; MacArthur, Christine; Magill, Laura; Morris, R. K.; Group, CHAPTER

This article was not externally reviewed at BMJ Open. Protocols that have been independently assessed prior to submission to BMJ Open are usually fast-tracked to publication on the grounds that further substantial changes will not be possible. This independent assessment will usually be external, independent review for both a substantial grant award from a non-commercial or government funder and ethics approval.
